# Development of a simplified smell test to identify Parkinson’s disease using multiple cohorts, machine learning and item response theory

**DOI:** 10.1038/s41531-025-00904-5

**Published:** 2025-04-23

**Authors:** Juan Li, Kelsey Grimes, Joseph Saade, Julianna J. Tomlinson, Tiago A. Mestre, Sebastian Schade, Sandrina Weber, Mohammed Dakna, Tamara Wicke, Elisabeth Lang, Claudia Trenkwalder, Natalina Salmaso, Andrew Frank, Tim Ramsay, Douglas Manuel, aSCENT-PD Investigators, Julianna J. Tomlinson, Natalina Salmaso, Brit Mollenhauer, Michael G. Schlossmacher, Brit Mollenhauer, Michael G. Schlossmacher

**Affiliations:** 1 Neuroscience Program, Ottawa Hospital Research Institute, Ottawa, ON Canada; 2 Methodological and Implementation Research Program, Ottawa Hospital Research Institute, Ottawa, ON Canada; 3 University of Ottawa Brain and Mind Research Institute, Ottawa, ON Canada; 4 Aligning Science Across Parkinson’s (ASAP) Collaborative Research Network, Chevy Chase, MD USA; 5 Department of Cellular and Molecular Medicine, University of Ottawa, Ottawa, ON Canada; 6 Department of Medicine, University of Ottawa, Ottawa, ON Canada; 7 Division of Neurology, Department of Medicine, The Ottawa Hospital, Ottawa, ON Canada; 8 Paracelsus-Elena-Klinik, Kassel, Germany; 9 Department of Neurology, University Medical Center Goettingen, Goettingen, Germany; 10 Department of Neuroscience, Carleton University, Ottawa, ON Canada; 11 Memory Program, Bruyère Research Institute, Ottawa, ON Canada; 12 The Methods Centre, Ottawa Hospital Research Institute, Ottawa, ON Canada; 13 School of Epidemiology and Public Health, University of Ottawa, Ottawa, ON Canada; 14 Deutsches Zentrum für Neurodegenerative Erkrankungen (DZNE), Goettingen, Germany

**Keywords:** Movement disorders, Neurodegenerative diseases, Parkinson's disease, Diagnostic markers, Movement disorders, Parkinson's disease

## Abstract

To develop a simplified smell test for identifying patients with Parkinson’s disease (PD), we reevaluated the Sniffin’-Sticks-Identification-Test (SST-ID) and University-of-Pennsylvania-Smell-Identification-Test (UPSIT), using three case-control studies. These included 301 patients with PD or dementia with Lewy bodies (DLB), 68 subjects with multiple-system atrophy (MSA) or progressive supranuclear palsy (PSP), and 281 healthy controls (HC). Scents were ranked by area-under-the-curve values for group classification and results leveraged by 8 published studies with 5853 individuals. PD/DLB patients showed markedly worse olfaction than controls, whereas scores for MSA/PSP subjects were intermediate. We identified and validated a subset of 7 shared odorants that performed similarly to the traditional 16-scent SST-ID and 40-scent UPSIT tests in distinguishing PD/DLB from HC. There, the identification of 4 or fewer scents out of 7 served as an effective cut-off between the two groups. We also identified a critical role for distractors (from correct answers) and age on olfaction performance.

## Introduction

Hyposmia is a common non-motor sign of Parkinson’s disease (PD) and dementia. The reported prevalence of olfaction loss in PD ranges from 45% to >90% based on populations selected, testing methods, and threshold criteria^1^. Chronic hyposmia is also viewed as predictive, with reduced olfaction preceding PD diagnosis by 4–20 years^1–3^. Olfactory testing may also help in the differentiation of parkinsonian syndromes^4^. Several screening tools and predictive models for the incidence of PD have included subjective or objective assessments of olfaction^5–9^.

Two commonly used smell tests for evaluating olfactory functions include the University of Pennsylvania Smell Identification Test (UPSIT)^10^ and the Sniffin’ Sticks Test (SST) battery^11,12^, comprising three subtests (i.e., for Identification (SST-ID), Discrimination (SST-DS), and Threshold (SST-TH)). The SST-ID and UPSIT are comparable in that they both assess one’s ability to identify a range of scents.

Smell test kits were initially developed to assess olfaction in the general population but have been increasingly used in research settings that study disorders of the brain. Using different cohorts and methods, some studies have ranked odorants in UPSIT^13–16^ and SST-ID^17–20^ by their diagnostic performances, and reported that certain subsets of scents appeared to have equal or better performance than the entire 40- or 16-scents-based tests. However, external validation was frequently missing in these analyses, proposed scent combinations were found to be cohort-specific without agreement across different studies^15,21^, and the role of distractors (versus the correct option for the scent tested) in such multiple-choice settings was understudied. Furthermore, analyses of UPSIT and SST-ID kits were always conducted separately, despite the similarities between the two tests. Finally, olfaction scores in patients with other, atypical forms of parkinsonism have not been assessed in PD-centric studies.

**Fig. 1:**
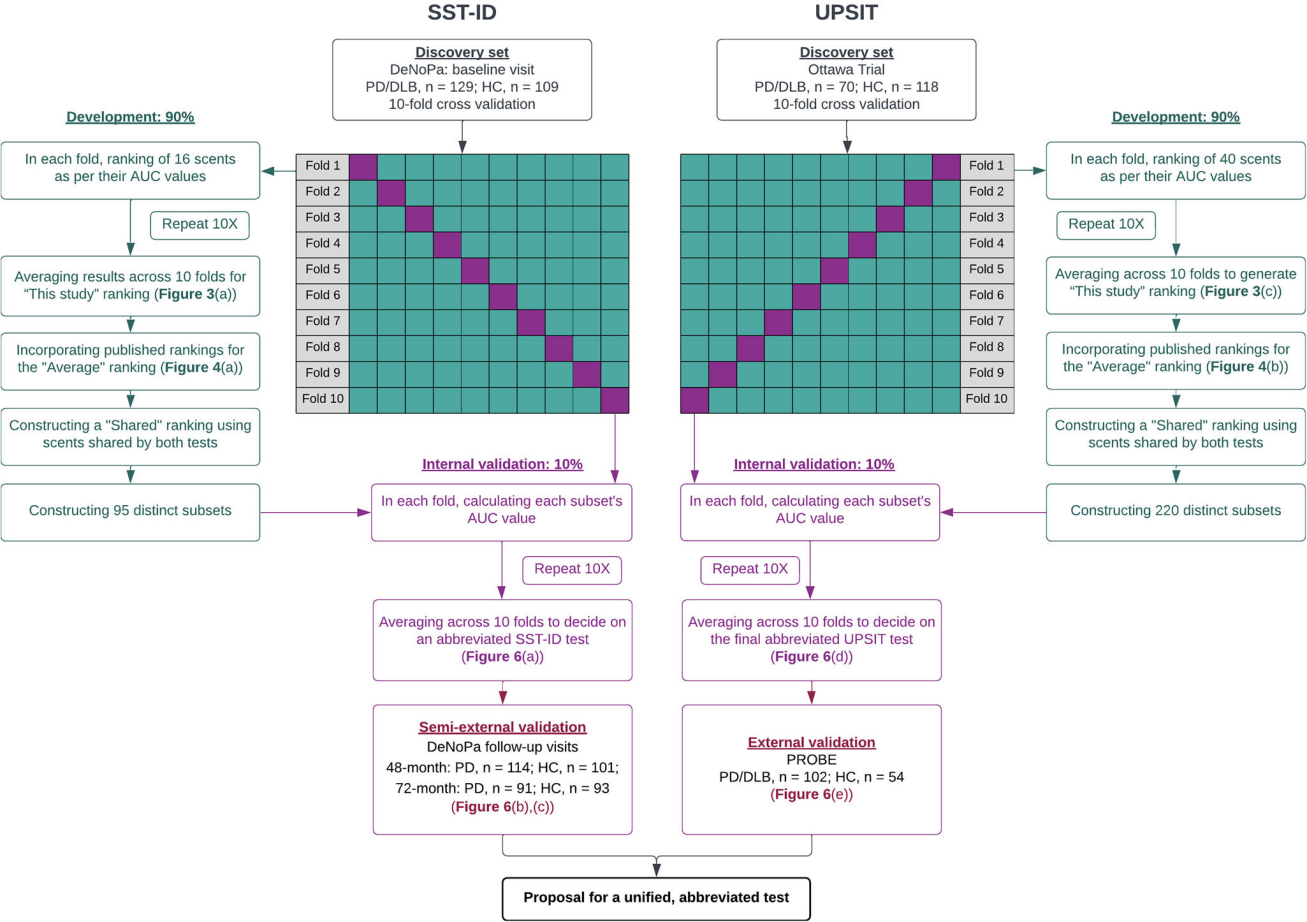
Machine learning workflow for developing and validating an abbreviated smell test for Parkinson’s disease. Details of the workflow are as indicated and described in Methods and Result sections of the main text. SST-ID Sniffin’ Sticks Identification test, UPSIT University of Pennsylvania Smell Identification Test, DeNoPa De Novo Parkinson Study, PROBE Prognostic Biomarkers in Parkinson Disease, HC healthy control, PD Parkinson disease, DLB dementia with Lewy bodies, MSA multiple system atrophy, PSP progressive supranuclear palsy, ROC receiver operating characteristic, AUC area under the ROC curve.

In this work, we aimed to assess olfaction performances in commonly encountered forms of parkinsonism; to assess individual features of both UPSIT and SST-ID odorants; to explain any observed differences of scent performance; and to develop a simplified smell test by unifying both kits using proper internal and external validation steps for the purpose of a potential screening tool. To this end, 8 published scent rankings^13–20^, collectively including 5853 participants, were incorporated into our study to make any proposed abbreviated test generalizable and to avoid overfitting. Further, we added Item Response Theory (IRT) based analyses^22^ to examine the behaviour of participants’ responses to multiple choices provided for each scent. Lastly, we analyzed the effects of age and sex on olfaction performance. Workflow of this study is illustrated in Fig. 1.

## Results

### Comparing different smell tests to classify typical Parkinson’s disease

We used de-identified data from three observational, retrospective, case-control studies: the De Novo Parkinson disease study (DeNoPa)^23^; the Ottawa (PREDIGT) Trial; and the Prognostic Biomarkers in Parkinson’s Disease Study (PROBE)^24^. Their demographic and diagnostic characteristics are summarized in Table 1.

**Table 1:** Baseline demographic characteristics and smell test performances for adults enrolled in three cohorts

Variable	DeNoPa					Ottawa Trial					PROBE				
	HC, N = 109^a^	PD/DLB, N = 129^a,d^	MSA/PSP, N = 9^a,d^	p-value^b^	q-value^c^	HC, N = 118^a^	PD/DLB, N = 70^a,e^	MSA/PSP, N = 6^a,e^	p-value^b^	q-value^c^	HC, N = 54^a^	PD, N = 102^a^	MSA/PSP, N = 53^a,f^	p-value^b^	q-value^c^
Sex				0.6	0.7				0.005	0.015				0.058	0.087
Female	42 (39%)	45 (35%)	2 (22%)			74 (63%)	29 (41%)	5 (83%)			28 (52%)	34 (33%)	18 (34%)		
Male	67 (61%)	84 (65%)	7 (78%)			44 (37%)	41 (59%)	1 (17%)			26 (48%)	68 (67%)	35 (66%)		
Age	65 (60, 70)	66 (58, 72)	72 (65, 76)	0.073	0.15	68 (58, 73)	68 (60, 74)	66 (63, 70)	0.9	0.9	59 (55, 69)	61 (55, 69)	67 (61, 75)	0.002	0.007
Parkinsonism duration at baseline in months	NA	14 (9, 24)	12 (6, 33)	0.7	0.7	NA	84 (36, 132)	48 (30, 66)	0.078	0.12	NA	65 (58, 71)	58 (57, 60)	0.2	0.2
Follow-up time in months	120 (120, 120)	120 (72, 120)	72 (48, 120)	0.001	0.004	ND	ND	ND			ND	ND	ND		
Smell test score^g^	12 (11, 14)	7 (4, 9)	10 (9, 11)	<0.001	<0.001	32 (29, 35)	17 (13, 22)	29 (26, 31)	<0.001	<0.001	35 (33, 37)	21 (14, 26)	28 (21, 32)	<0.001	<0.001
Smell test percentile^h^	50 (25, 75)	4 (4, 10)	25 (18, 50)	<0.001	<0.001	39 (18, 66)	6 (4, 10)	23 (14, 37)	<0.001	<0.001	56 (27, 73)	5 (4, 17)	23 (9, 42)	<0.001	<0.001
Olfaction^i^				<0.001	<0.001				<0.001	<0.001				<0.001	<0.001
Normal	94 (86%)	30 (23%)	7 (78%)			93 (79%)	7 (10%)	3 (50%)			50 (93%)	29 (28%)	34 (64%)		
Hyposmia/anosmia	15 (14%)	99 (77%)	2 (22%)			25 (21%)	63 (90%)	3 (50%)			4 (7%)	73 (72%)	19 (36%)		

*DeNoPa* De Novo Parkinson Study, *PROBE* Prognostic Biomarkers in Parkinson Disease, *IQR* interquartile range, *HC* healthy control, *PD* Parkinson disease, *DLB* dementia with Lewy bodies, *MSA* multiple system atrophy, *PSP* progressive supranuclear palsy, *NA* not applicable, *ND* not determined, *SST-ID* Sniffin’ Sticks Identification test, *UPSIT* University of Pennsylvania Smell Identification Test.

^a^*n* (%); Median (IQR).

^b^Fisher’s exact test; Kruskal–Wallis rank sum test.

^c^False discovery rate correction for multiple testing.

^d^PD: n = 126; DLB: n = 3; MSA: n = 4; PSP: n = 5

^e^PD: n = 69; DLB: n = 1; MSA: n = 5; PSP: n = 1

^f^MSA: n = 27; PSP: n = 26.

^g^SST-ID scores (0–16) for DeNoPa, UPSIT scores (0–40) for Ottawa Trial and PROBE.

^h^Age- and sex-adjusted normalized percentiles.

^i^Hyposmia/anosmia was determined by SST-ID percentile ≤10%, and UPSIT percentile ≤15%.

**Fig. 2:**
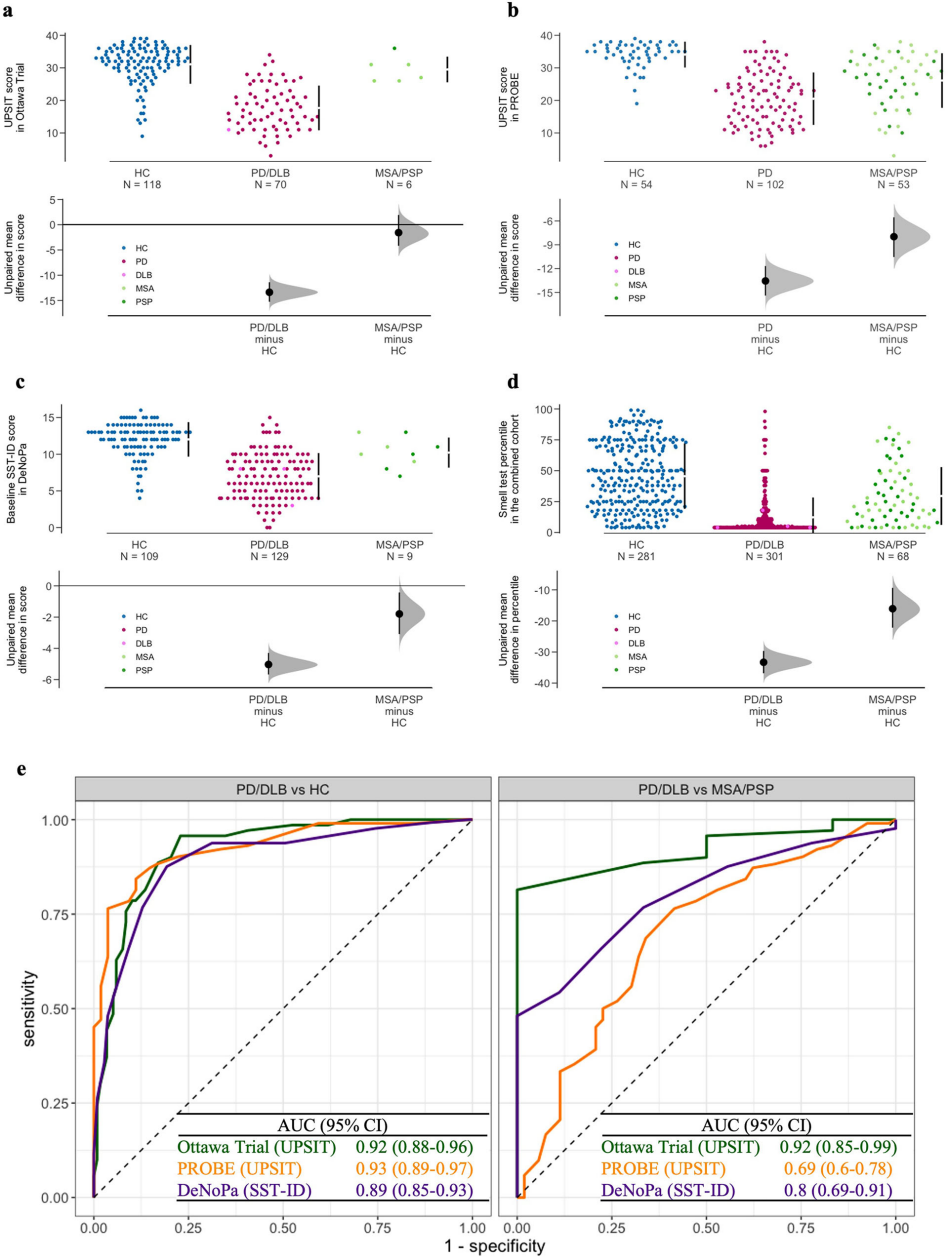
Distribution of olfaction scores using two established tests for different diagnostic groups with parkinsonism in three cohorts. Cummings estimation plots (**a**–**d**) were used to illustrate and compare smell test score distributions in each diagnostic group: **a** for UPSIT in the Ottawa Trial cohort, **b** for UPSIT in the PROBE cohort, **c** for SST-ID in the DeNoPa cohort, **d** UPSIT and SST-ID scores were transformed to percentiles based on age- and sex-adjusted norms in the combined cohorts. Each data point in the upper panels represents the score of one participant, and colors represent different groups and diagnosis, as shown in legends. The vertical lines in the upper panels represent the conventional mean ± standard deviation error bars. The lower panels show the mean group difference (the effect size) and its 95% confidence interval (CI) estimated by bias-corrected and accelerated bootstrap, using healthy controls as the reference group. **e** Shows ROC curves and AUC values with 95% confidence interval (CI) for smell tests in each cohort (indicated by different colors; individual scores shown in **a**–**d**) to distinguish PD/DLB versus HC groups (left) and PD/DLB versus MSA/PSP groups (right). Abbreviations as in Fig. 1.

As expected, across all three cohorts, PD and dementia with Lewy bodies (DLB) patients generally had lower smell test scores (i.e., worse olfaction) than neurologically healthy controls (HC), whereas scores for multiple system atrophy (MSA) and progressive supranuclear palsy (PSP) patients were intermediate. Their score distributions are shown in Fig. 2a–d, and median scores/percentiles as well as the percentages of hyposmia are listed in Table 1. There was no detectable difference in olfaction performance between MSA and PSP patients (Fig. 2b, d). UPSIT and SST-ID kits showed comparable performances in distinguishing PD/DLB patients from HC subjects (Fig. 2e, left) with area under the receiver operating characteristic (ROC) curve (AUC) values in the three cohorts ranging between 0.89 and 0.93.

Further, both tests showed reduced performance when comparing PD/DLB patients to MSA/PSP patients (Fig. 2e, right), but with a larger variation in AUC values (0.69–0.92) across the three cohorts due to the smaller sample sizes of MSA/PSP groups in the Ottawa Trial and DeNoPa cohorts. Furthermore, AUC values of UPSIT and SST-ID to differentiate PD/DLB patients from the combination of healthy controls and MSA/PSP patients are listed in Supplementary Table 1.

Among the three SST subtests, SST-ID was found to be the best in distinguishing PD/DLB patients from HC subjects as well as from individuals with MSA/PSP (Supplementary Fig. 1). For cohort-specific thresholds and corresponding sensitivity and specificity values, see Supplementary Table 1.

### Performances by individual scents differ in discriminating PD/DLB from healthy controls

**Fig. 3:**
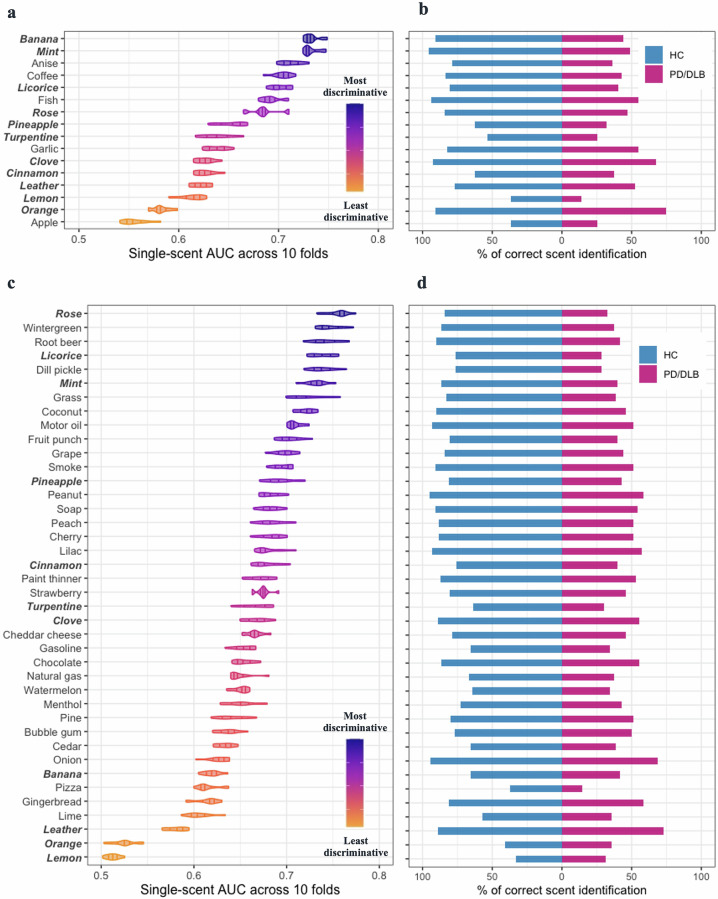
Individual scent performances in differentiating PD/DLB from healthy control groups. SST-ID scents are shown using baseline DeNoPa data (**a**, **b**) and UPSIT scents for the Ottawa Trial cohort (**c**, **d**). **a**, **c** illustrate the distribution of AUC values of each scent across 10-fold cross-validation using violin plots, with 25%, 50%, and 75% quantile lines. The scents are ordered in descending order of their mean single-scent AUC value (top to bottom); the color of each scent changes gradually from the most to the least discriminative value, as indicated by the legend. Scents shared by both tests are highlighted in bold italic font. **b**, **d** Shows the percentage of subjects correctly identifying each scent within both groups in each corresponding cohort. Abbreviations as in Fig. 1.

Figure 3a shows the distribution of AUC values for each SST-ID scent across 10 folds using baseline data from the DeNoPa cohort. Clusters of scents identified included *banana* and *mint* as the two most discriminative scents (individual AUC values, ≥0.725), followed by *anise*, *coffee*, *licorice*, *fish*, and *rose* in the second-most discriminative cluster. Compared with SST-ID scents, clustering was less obvious for the UPSIT scents (Fig. 3c), where AUC values ranged between 0.5 to 0.77. In the Ottawa Trial cohort, the top-ranked 7 UPSIT scents in identifying established PD/DLB patients vs. controls included *rose*, *wintergreen*, *root beer*, *licorice*, *dill pickle*, *mint*, and *grass*.

The observed differences in each scent’s discriminative performance were further examined by visualizing the percentages of correct scent identification within each diagnostic group (Fig. 3b, d) and by the percentage differences between HC and PD/DLB groups (Supplementary Fig. 2). Regardless of the study cohort and smell test used, PD/DLB patients showed lower percentages of correctly identifying each scent than control subjects. Scents that were easy to identify in the HC group but difficult for the PD/DLB group (i.e., generating larger percentage differences, as shown in Supplementary Fig. 2) had greater single-scent AUC values. Scents had poorer discriminative performances when both groups found them easy (e.g., in SST-ID: *orange*; UPSIT: *leather*) or difficult (e.g., in SST-ID: *apple*; UPSIT: *lemon*) (Fig. 3b, d).

**Fig. 4:**
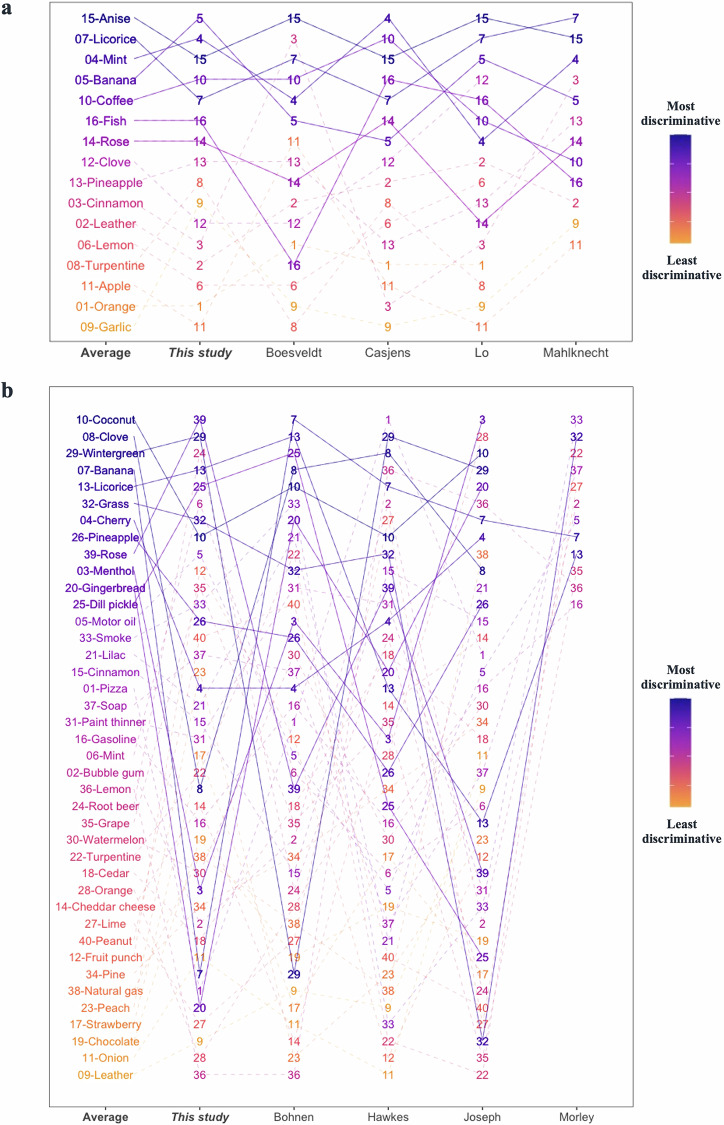
Comparison of scent rankings in this study versus previously published ones. **a**, **b** show scent rankings of SST-ID and UPSIT, respectively. “This study” columns show scent rankings from Fig. 3, and the neighboring columns show corresponding rankings from other studies, as indicated on the x-axis. The “Average” column of each panel shows the scent ranking generated by averaging results from 5 separate rankings. Each scent is represented using the format “index-scent” in the “Average” ranking, and as index only in others. The lines track how each scent’s rank changes from study to study. Color of each scent changes gradually from the most to the least discriminative odorant defined by “Average”. Based on these, the 7 best-performing scents in SST-ID (**a**) and the 12 best-performing scents in UPSIT (**b**) are tracked by solid lines. Note, rankings by Mahlknecht et al. and Morley et al. included only the top 12 scents.

Therefore, rankings for scents used in the SST-ID and UPSIT kits were constructed. Figure 4 compared the scent rankings from this study with previously published reports, and as a result, two “Average” rankings were derived. For the SST-ID kit, several studies -despite the differences in cohort design and methods applied (Supplementary Table 2)- showed consensus that *anise*, *licorice*, *mint*, *banana*, *coffee*, *fish*, and *rose* were the most discriminative scents in distinguishing PD/DLB subjects from HCs (Fig. 4a). For the UPSIT battery, however, related studies generated less agreement on scent rankings (Fig. 4b), which could be partially explained by results shown in Fig. 3c. There, many UPSIT-based scents showed similar performances (amongst each other), and therefore, they revealed fewer clusters than did SST-ID-based scents. Nonetheless, the top-ranked 7 UPSIT scents in the final “Average” list included: *coconut*, *clove*, *wintergreen*, *banana*, *licorice*, *grass*, and *cherry*. Because there are 11 scents shared between SST-ID and UPSIT kits, an additional “Shared” ranking was generated by us to construct a potentially unified, abbreviated smell test (Supplementary Table 3).

### IRT analysis reveals further scent details and the influence of distractors

**Fig. 5:**
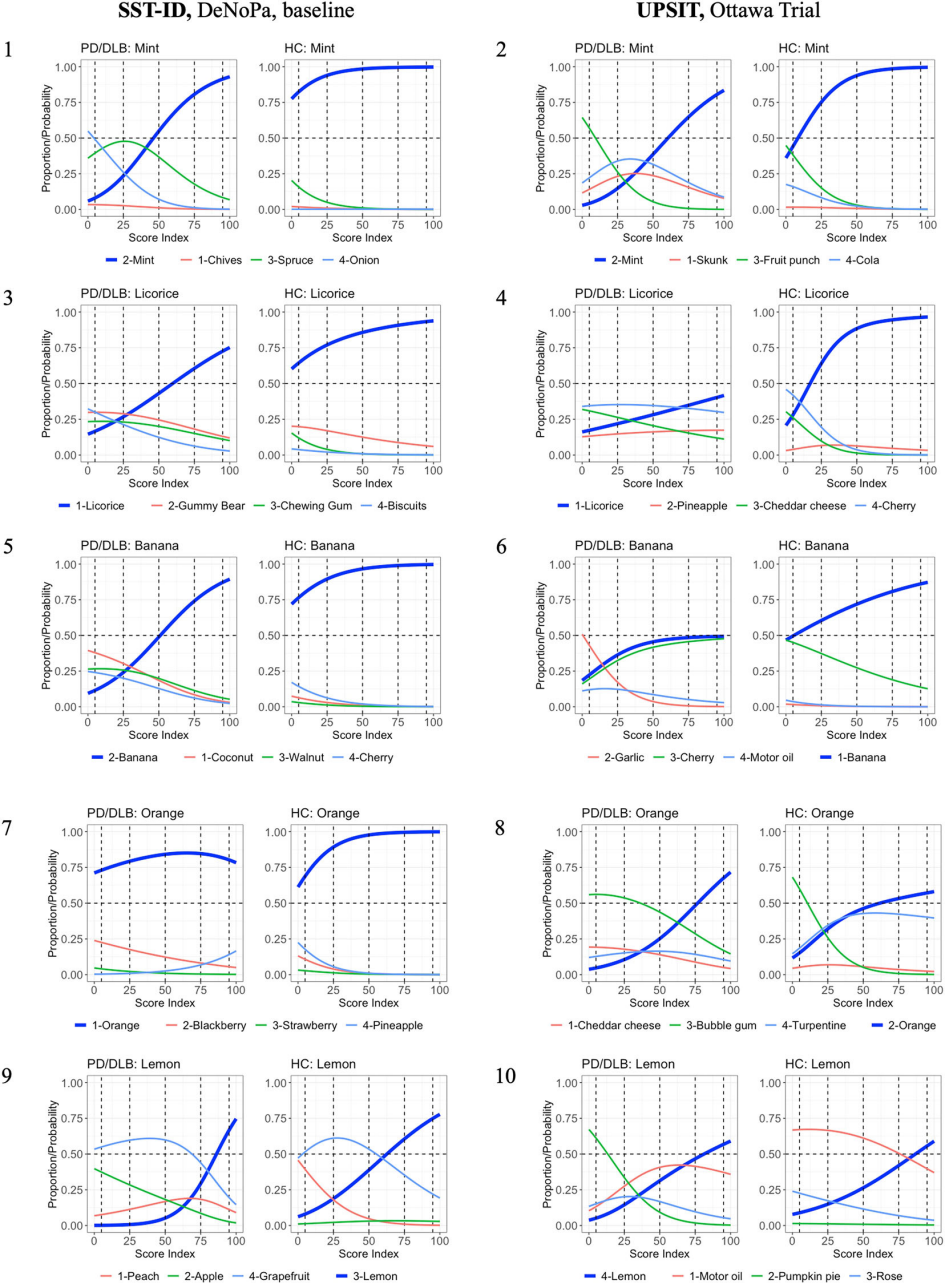
Influence of distractors in multiple-choice smell tests for five shared scents selected. Panels with odd numbers show the Item Characteristic Curves (ICCs) of five SST-ID scents: *mint*, *licorice*, *banana*, *orange*, and *lemon*. Panels with even numbers show ICCs of the corresponding UPSIT scents. In each figure, panels on the left show data for PD/DLB patients, panels on the right for healthy controls (HC). The x-axis reveals transformed score indices in [0,100] (percentage rank of the respective scores) within the corresponding group. The y-axis shows the probability of choosing each option at a particular score index. The correct option of each item is highlighted using thicker, blue curves. Numbers in the color legends represent option indices. The horizontal dashed lines represent 50% probability. The vertical dashed lines represent five quantiles (5%, 25%, 50%, 75%, and 95%).

In the current context, *mint* and *licorice* were two well-performing scents. Hence, using IRT analysis, their Item Characteristic Curves (ICCs) (Fig. 5(1)–(4)) showed similarities in that HC subjects generally correctly identified them, while PD/DLB patients had more difficulty in choosing the correct option. However, there were also some noteworthy differences. When scoring on the scent presented for *mint*, PD/DLB patients could rule out ‘chive’ and ‘onion’ in the SST-ID assay and ‘fruit punch’ in UPSIT, indicating that they detected some scent, but it was not declarative enough for subjects to correctly choose *mint*. However, for *licorice*, particularly in the UPSIT kit, there was strong evidence of random guessing whereby patients couldn’t detect any scent to help favor or eliminate an option (Fig. 5(4), left). Here, ICCs of scoring by HCs also eliminated the possibility of the corresponding pen (SST-ID) or encapsulated patch (UPSIT) being defective.

Curiously, the scent for *banana* was discriminative in DeNoPa but not in the Ottawa Trial (Fig. 3); these inconsistent performances were not due to differences in distractors. When testing for *banana*, the option ‘cherry’ distracted many PD/DLB patients and HCs in the Ottawa Trial, but not in the DeNoPa study (Fig. 5(5)-(6)). Here, cohort-specific or odorant-related differences (*e.g*., the concentration or composition for the artificial scent offered) might offer more plausible explanations.

*Orange* and *lemon* were both ranked low in the two tests but for different reasons (Fig. 5(7)–(10)): *orange* in SST-ID was relatively easy, even for hyposmic PD/DLB patients. *Orange* in UPSIT, however, had different distractors that were active within PD/DLB (‘bubble gum’) and HC (‘turpentine’) groups. For *lemon*, the distractors of ‘grapefruit’ in SST-ID and ‘motor oil’ in UPSIT confused both patients and healthy persons. Such ICC results within the normosmic control group (Fig. 5(8)–(10), right) might be evidence of a flawed odorant/distractor choice or an explanation that is rooted in chemical manufacturing of the scent. The ICCs for all other scents are shown in Supplementary Figs. 3–5.

### Development and validation of abbreviated smell tests

**Fig. 6:**
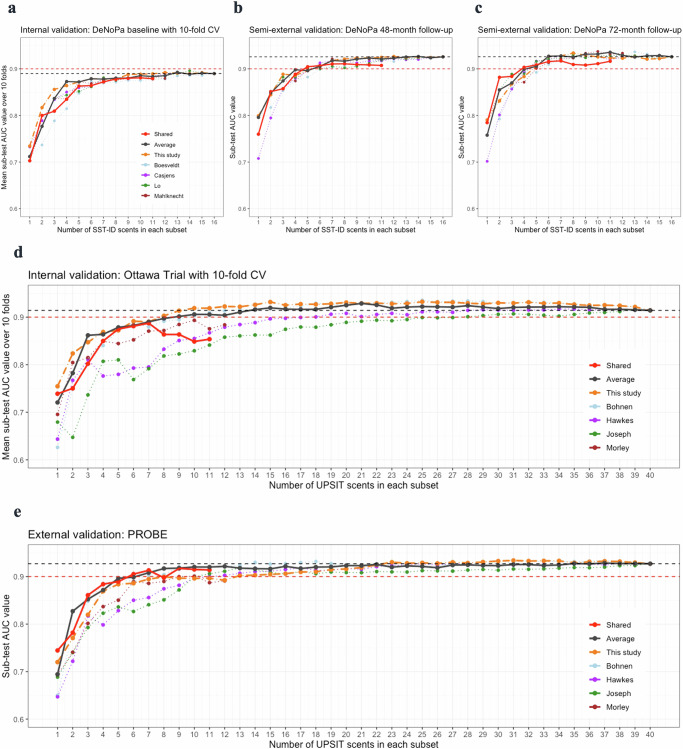
Exploration of smaller subsets of scents tested in their accuracy of group classification for PD/DLB subjects versus healthy controls. The x-axis shows the number of individual scents used for each subset examined; colors represent different scent rankings from separate studies, as indicated by the legends (see also Fig. 4 and Supplementary Table 3). ‘Shared’ denotes scents used in both UPSIT and SST-ID; ‘Average’, all studies combined; ‘This study’, rankings derived using baseline DeNoPa and Ottawa Trial data. Individual points shown in **a**, **d** represent internal validation results, averaging across 10 folds. **b**, **c**, **e**, each point represents the AUC value of the corresponding subset using (semi-)external validation sets. The black horizontal, dashed lines indicate AUC values of the corresponding test when viewed in its entirety. Red horizontal, dashed lines indicate AUC = 0.9 as a predetermined reference line.

Figure 6 visualizes AUC values of all subsets of scents examined within internal and external validation datasets (as summarized above in Fig. 1). When using an increasing number of highly rank-ordered scents, we observed that the corresponding AUC values for odorant subsets increased steeply for the first four, indicating that these more discriminative scents were complementary to each other and not redundant (Fig. 6). Surprisingly, any improvement in subset performance thereafter was marginal. When compared with other published rankings, the “Average” rankings as well as their subsets appeared to be more discriminative with robust performances in all the validation datasets. Considering a balanced trade-off between the number of scents administered and subset performance, an SST-ID version with just 7 scents (shown in Fig. 6a–c) and an UPSIT version of 10 scents (Fig. 6d, e) emerged as the best performers in this analysis, as highlighted by the corresponding “Average” rankings (shown as black lines).

### Development of an integrated smell test to differentiate PD and DLB from healthy subjects

To develop a potentially unified smell test, we found that the subset of 7 scents from the “Shared” ranking between SST-ID and UPSIT kits (red lines in Fig. 6, Supplementary Table 3) with the highest performance in all validation datasets comprised *licorice*, *banana*, *clove*, *rose*, *mint*, *pineapple*, and *cinnamon*. When combining all three studies (DeNoPa; Ottawa Trial; PROBE), this abbreviated test of 7 odorants could distinguish PD/DLB patients from healthy subjects with an AUC value of 0.87 (95% confidence interval (CI) 0.85-0.9). Under these circumstances, the correct identification of 4 or fewer scents out of 7 tested served as an effective cut-off to distinguish between the two groups (Table 2). Median (interquartile range (IQR)) scores, AUC values, thresholds and the associated sensitivity and specificity results for these subsets within each individual trial as well as within the combined cohort are shown in Table 2. It also shows the 7-scents’ performance in differentiating PD/DLB patients from all other subjects (i.e., the combination of healthy controls and MSA/PSP subjects) with nearly identical AUC values as well as for the cut-off of ≤4 scents to separate them.

**Table 2:** Performance of the 7-scent abbreviated test (score range: 0-7) in distinguishing PD/DLB patients from controls

Cohort	Median (IQR)			PD/DLB vs HC				PD/DLB vs Other (HC and MSA/PSP)			
	HC	PD/DLB	MSA/PSP	AUC (95% CI)	Threshold^a^	Sensitivity	Specificity	AUC (95% CI)	Threshold^a^	Sensitivity	Specificity
*Baseline visit*											
Combined	6 (5, 7)	3 (2, 4)	5 (3.75, 6)	0.87 (0.85–0.9)	≤4	0.76	0.85	0.85 (0.82–0.88)	≤4	0.76	0.8
DeNoPa	6 (5, 6)	3 (2, 4)	5 (4, 5)	0.88 (0.83–0.92)	≤4	0.76	0.84	0.87 (0.82–0.91)	≤4	0.76	0.82
Ottawa trial	6 (5, 7)	3 (2, 4)	5.5 (4.25, 6)	0.89 (0.84–0.93)	≤4	0.8	0.81	0.88 (0.84–0.93)	≤4	0.8	0.8
PROBE	7 (6, 7)	3 (2, 4.75)	6 (3, 6)	0.91 (0.87–0.96)	≤4	0.75	0.98	0.8 (0.73–0.86)	≤4	0.75	0.79
*Follow–up visits*											
DeNoPa, 48–month	6 (5, 7)	3 (1, 4)	5 (3, 6)	0.91 (0.87–0.95)	≤4	0.83	0.87	0.9 (0.86–0.94)	≤4	0.83	0.85
DeNoPa, 72–month	6 (5, 7)	2 (1, 4)	5 (5, 6)	0.91 (0.86–0.95)	≤4	0.87	0.86	0.91 (0.87–0.95)	≤4	0.87	0.86

*DeNoPa* De Novo Parkinson Study, *PROBE* Prognostic Biomarkers in Parkinson Disease, *IQR* interquartile range, *HC* healthy control, *PD* Parkinson disease, *DLB* dementia with Lewy bodies, *MSA* multiple system atrophy, *PSP* progressive supranuclear palsy, *CI* confidence interval.

^a^Note, for the 7scent test, DeNoPa, PROBE and the combined cohort generated a consensus of 4 as the optimal threshold. In the Ottawa trial, the optimal threshold corresponding to the maximum Youden Index was 5, with a sensitivity of 0.97 and a specificity of 0.66 for PD/DLB vs HC, and a sensitivity of 0.97 and a specificity of 0.65 for PD/DLB vs Other.

### Performances of scents in the differentiation of PD from MSA and PSP

The following analysis for the comparison between patients with PD and MSA/PSP focused on the participants in the PROBE study; the other two cohorts (DeNoPa; Ottawa Trial) had too few MSA/PSP subjects to reliably analyze the performance of any scent subset. In PROBE, the same list of the 7 top-ranked, shared scents distinguished patients with PD from those with MSA/PSP at an AUC value of 0.68 (95% CI 0.58–0.77), which was similar to the AUC value using the complete 40-scent UPSIT kit (0.69 (95% CI 0.6–0.78), see Supplementary Table 1).

However, to determine whether this separation could be improved, the same workflow from above, including for validation steps, was applied to the PROBE cohort to potentially generate a subset of scents more specific for the distinction of PD vs. MSA/PSP patients. Intriguingly, a subset of 10 scents (*clove, dill pickle, cinnamon, soap, rose, pizza, root beer, turpentine, gasoline*, and *licorice*) achieved the highest value, i.e., an average AUC of 0.78 (95% CI 0.52–0.99) in the validation set for PROBE, or 0.77 (95% CI 0.69–0.85) in the entire cohort. Hence, this outcome represented an improvement when compared to the entire 40-scent UPSIT kit (Supplementary Fig. 6). Of note, adding additional scents above 10 did not substantively increase the degree of separation between the PD and MSA/PSP groups (Supplementary Fig. 6). Using a cut-off for 6 or fewer correctly identified odorants (out of these 10 top-ranked scents tested) separated PD patients from MSA/PSP subjects in the PROBE cohort with a sensitivity of 0.77 and specificity of 0.68.

### Assessment of age and sex on scent identification

We also investigated the influence of age and sex on olfaction performance. Supplementary Table 4 shows the coefficients of the linear regression for the relationship between smell test scores with age, sex, and diagnostic groups within each cohort. Not surprisingly, progression in age significantly lowered olfaction across all groups. In addition, males generally showed a worse sense of smell than their female counterparts, although the latter was not significant across the three cohorts.

When focusing just on the 11 scents shared between SST-ID and UPSIT kits, relationships between scent identification and sex were further evaluated by comparing the percentages of correct scent identification across groups (Supplementary Fig. 7a). In line with the regression results, females showed higher percentages of correct identifications than males for most of the scents, except for *cinnamon*, *turpentine*, and *leather*.

Finally, we compared the probability of identifying each scent correctly across ages between the PD/DLB and HC groups (Supplementary Fig. 7b). As anticipated, older participants generally showed decreasing percentages for correctly identifying specific odorants. The fitted lines for PD/DLB and HC groups were usually in the same direction and of similar slopes, with some exceptions, but these were not consistent across all three cohorts.

## Discussion

To our knowledge, this is the most comprehensive study to date describing olfactory dysfunction in late-onset, typical PD and two less frequent forms of parkinsonism using both SST and UPSIT. When probing for hyposmia in PD, the following points seem to matter: PD/DLB patients had worse olfaction than healthy subjects, and scores of MSA/PSP patients were intermediate without a detectable difference between them; when screening populations for PD using SST, scent identification testing is sufficient, and the threshold and discrimination subtests could be omitted; fewer scents can reduce examination time and test taking fatigue without sacrificing diagnostic accuracy; the selection of fewer scents should be informed by their discriminative performance in specific group classification efforts; random guessing lowers diagnostic accuracy; and from a test design perspective, choices provided as distractors influence scent identification performance. Importantly, we found that an abbreviated smell test -created by carefully selecting ‘specific scents’- is sensitive enough to identify PD/DLB-linked hyposmia. Such a simplified test, which is now being piloted by us prospectively, holds the potential to facilitate olfactory testing in the outpatient clinic setting, for at-home testing and in population-based screening efforts.

In developing and validating an abbreviated smell test, we used a machine learning approach (Figs. 1, 3, 4, 6) and found that a set of only 7 scents (*licorice*, *banana*, *clove*, *rose*, *mint*, *pineapple*, and *cinnamon*) was sufficient to approximate the diagnostic performance of administering either the complete 16-scent SST-ID or 40-scent UPSIT batteries, and that the value of adding more scents was negligible (Fig. 6).

We also demonstrate the impact distractors have on detecting individual scents using IRT analysis (Fig. 5, Supplementary Figs. 3–5). We uncovered uncertainty in eliciting a choice for some scents, even for healthy subjects with intact olfaction. This could be explained by the difficulty of biological scent discrimination or a to-be-improved selection of artificial odorants. By extension, our analyses revealed the opportunity to remove ill-performing scents, e.g., *orange* and *lemon*, from currently used kits.

Of general importance, we found a high level of guessing among PD patients for some scents, indicating patients’ difficulty in detecting them. SST-ID and UPSIT batteries are multiple choice-based tests, in which participants are instructed to always choose one answer even when they cannot smell anything; such random guessing will introduce errors into data sets. Advanced IRT methods can treat missing responses as an additional option; administrators of tests would then prefer the participants to leave any uncertain questions unanswered rather than forcing a guess. However, for the future administration of standardized olfaction tests, or for designing a new one, including an abbreviated one, we suggest adding an extra choice, such as “*I cannot identify the scent*” to reduce random guessing. Based on our experience in administrating smell tests, the extra option would also help improve participant experience and eliminate frustration in patients with severe hyposmia.

Our goal in creating a simplified smell test was for it to be used in the future as a screening tool to identify patients with probable PD, but it should not replace UPSIT and SST for other purposes without robust testing. The cut-offs reported in Table 2 were for group classification; these do not represent cut-off values for diagnosing an individual’s hyposmia or anosmia. More data, preferably obtained from the general population, are needed to compare scores of a simplified smell test to those from UPSIT/SST-ID kits and to establish cut-offs in order to diagnose hyposmia or anosmia; further, the influence of age and sex should be considered whenever possible. This can be achieved through simulation within UPSIT/SST-ID data sets like in our study, or more robustly, in specifically designed trials where participants are assessed by both, a routinely used test (such as SST-ID or UPSIT) and an abbreviated version, to permit a head-to-head comparison. We have recently begun such an effort at three separate clinic sites. Of note, the cut-off values listed in Table 2 and Supplementary Table 1 were associated with maximum Youden Indices, while in practice, cut-offs may also be determined based on specific study purposes.

As a screening tool, an easy-to-administer, inexpensive, sensitive and non-invasive smell test (such as one that is based on 7 scents) could have important usefulness, particularly when coupled with a short, self-administered questionnaire capturing demographic information and known risk factors of developing PD^9^. Such a questionnaire may also identify factors leading to hyposmia unrelated to neurodegeneration, e.g., previous nasal injuries, microbial infections, seasonal allergies, and chronic exposure to air pollution, to augment specificity for PD. Upon validation, such a kit could be used as the initial step of large-scale community screening, or in routine neurological practice of a movement disorders-oriented clinic, or for early detection within a family medicine office. When it comes to screening efforts for typical PD, more invasive and expensive tests, *e.g*., the α-synuclein seeding amplification assay from cerebrospinal fluid (CSF) or skin biopsies, or the administration of a dopamine transporter scan, could be employed as additional steps to increase diagnostic accuracy, such as when aiming to enroll subjects with probable PD into specific, disease-modifying trials^25–29^.

Among the three SST subtests, SST-ID performed the best in distinguishing PD/DLB patients from healthy controls (and from MSA/PSP patients) and therefore was the focus of this study. However, the other two subtests could be useful in other scenarios: SST-DS has been shown to have stronger correlation with disease duration than SST-ID^17^, and SST-TH performed better in separating the akinetic-rigid dominant and tremor-dominant subtypes^30^. Further, all three subtests may be needed for the most comprehensive assessment of one’s olfaction^31^. Hence, the choice of appropriate subtest(s) will always be selected based on the specific research question.

Despite the findings regarding scent ranking and subset analyses, it remains unclear whether a specific PD olfaction deficit exists, rather than a global reduction in scent processing, and what the underlying mechanisms could be. We and others recently found that chronic hyposmia and anosmia were significantly associated with positivity on the α-synuclein seeding amplification assay in CSF, suggesting that patients may have an underlying disease linked to the dysregulation of *SNCA* expression and/or protein processing^32,33^.

Mechanistically, it not only remains unknown as to how chronically reduced olfaction arises in PD/DLB (and REM Sleep Behaviour Disorder) as well as some MSA/PSP subjects, but also at what age it begins, at what site within the olfactory circuitry, and whether hyposmia is shared for specific scents among persons with PD/DLB versus those with dementia syndromes unrelated to a synucleinopathy disorder. Large scale population screening efforts, including with a simplified testing battery derived from SST-ID and UPSIT kits, could begin to answer these questions.

One limitation of our study is the small sample size of patients diagnosed with MSA or PSP, two much less frequent variants of parkinsonism, especially in the DeNoPa and Ottawa Trial cohorts. The scent ranking and associated subset developed here for distinguishing PD versus MSA/PSP patients therefore represent preliminary results, and more data are needed for further validation, such as by combining multiple small cohorts. Distinguishing these different forms of parkinsonism early in their course based on inexpensive, simple-to-test biomarkers would be of great value.

Another limitation of our study includes the fact that the cohorts examined here are highly homogeneous with most participants being White. Although scent rankings and the selection of a simplified smell test have been rigorously developed and validated with external information incorporated, future calibration and cultural adaption efforts will be necessary when testing other populations, including of greater ethnic diversity. To this end, an integrated, simplified test is currently being piloted at two sites in North America and one site in Europe to compare its performance with UPSIT and SST-ID batteries, respectively, using four different languages.

Further, case-control studies have an inherent potential for selection bias in their recruitment. Especially because of their design, age- and sex-effects were likely underestimated in our cohorts. Population studies, such as in community screening efforts undertaken previously by PARS planners^34^ or with ‘PPMI Remote’ by the Parkinson’s Progression Markers Initiative (PPMI) study^35^, could provide complementary data sets. However, these have potential setbacks as well: As the majority of participants will have a normal sense of smell, score distributions could be skewed; and if smell test data are reduced to a single sum score (rather than the detailed response to each scent), sub-analyses will be difficult to complete, thus limiting interrogations of data sets between different cohorts.

Last-but-not-least, for screening purposes a one-time administered smell test may not be informative enough to assess a subject’s sense of smell completely, because other factors, such as temporary hyposmia due to an upper respiratory tract infection, seasonal allergies, occupational exposure and/or due to beverage consumption, eating, smoking before taking the test, could skew results. Retesting at appropriate time intervals, as was carried out in the DeNoPa Study at predetermined time points, may be required for even higher accuracy in performance (Table 2). Such efforts will be facilitated by an inexpensive, easily administered, abbreviated, yet sensitive smell test that ensures completion and reduces random guessing when providing answers.

## Methods

The study was conducted in adherence with the STARD^36^ guideline (see Supplementary Table 5).

### Source of data and participants

We used de-identified data from three observational, retrospective, case-control studies: DeNoPa^23^; the Ottawa (PREDIGT) Trial; and PROBE^24^. Their demographic and diagnostic characteristics are summarized in Table 1. Data of the cross-sectional Ottawa Trial study, baseline data of the longitudinal PROBE study, and three visits of the longitudinal DeNoPa study (baseline; 48-month; and 72-month follow-up visits) were used. Patients with PD, DLB, MSA, or PSP, and neurologically healthy controls were included (Table 1). Most study participants with PD in the three cohorts were classified as Hoehn-and-Yahr stage II-III. No participant overlap existed between the three studies.

The DeNoPa cohort^23^ is an ongoing, single-center study based in Kassel, Germany. It is an observational, longitudinal study of patients with a newly established diagnosis of PD (UK Brain Bank Criteria^37^), who were naïve to L-DOPA therapy at baseline, and of age- and sex- and education-matched, neurologically healthy controls. Details of inclusion/exclusion criteria have been described elsewhere^23^. Diagnostic accuracy was ensured by ongoing follow-up visits every two years (as of 2023, 10-year follow up visits were underway). Consequently, diagnosis of 12 patients were later updated as DLB, MSA, or PSP (see Table 1). Data used were received on May 16th, 2023.

The Ottawa Trial is a pilot study to evaluate the performance of a 2-step screening tool that combines the PREDIGT questionnaire^8,9^ and the UPSIT test to distinguish patients with PD/DLB from age-matched neurologically healthy controls and patients with various other neurological diseases. Enrolment and assessment of this cross-sectional, case-control study was completed in March 2024. A manuscript that describes this cohort is in preparation. Diagnostic accuracy was ensured by independent chart review by three subspecialty-trained neurologists according to UK Brain Bank Criteria^37^ and MDS Criteria^38^.

PROBE^24^ is a longitudinal, case-control study to test biomarkers in PD subjects and various controls to determine their feasibility and potential utility as markers of risk and prognosis for PD. Details of inclusion/exclusion criteria have been described elsewhere^24^. Participants were enrolled from August, 2007 to December, 2008. The diagnoses of PD, probable MSA, and probable PSP were established using UK Brain Bank criteria^37^, Consensus Criteria^39^, and NINDS-PSP Criteria^40^, respectively.

Analyses of deidentified cohort data were approved by Investigational Review Boards at Paracelsus-Elena-Klinik (Kassel) in Frankfurt, Hesse, Germany (FF 89/2008), the Ottawa Hospital (Ottawa, Ontario, Canada; 20180010-01H), and all PROBE Study-affiliated sites in North America, with participants’ consent.

### Study assessments

The SST comprises a supervised test administered in clinic settings using pen-like odor dispensing devices^11,12^. It has three subtests: SST-ID, SST-DS, and SST-TH, each with 16 odorants. In SST-ID, subjects are presented a stick and choose the scent from four options. SST-DS is performed using triplets of odorants that are of similar intensity and hedonic tone, where subjects are required to identify which stick of the triplet has a different scent from the other two. SST-TH is performed using triplets of sticks where only one is filled with odorant at a certain dilution whereas the other two are filled with odor-free solvent. SST-TH determines at what dilution subjects can consistently identify the odorant-filled stick. The entire SST (in German) was completed by all DeNoPa participants at their baseline visits, and the SST-ID subtest was re-administered at 48-month and 72-month follow-up visits.

UPSIT was used in the Ottawa Trial and PROBE; this self-administered kit (in English) contains 40 scratch-and-sniff questions, presented as multiple-choice responses, with 4 options offered for each scent^10^.

### Data preparation and analysis

Observations that had no valid SST-ID/UPSIT response were removed. Observations with incomplete responses were imputed with 0s, indicating incorrect responses. A dichotomous response-based transformation (0 = incorrect; 1 = correct) was used to calculate the sum scores and assess discriminative performances for each scent. The exact indices of chosen options were used for IRT analysis.

Demographic and diagnostic characteristics of the study cohort were summarised using n (%), and median (IQR). The reported p-values represented the significance from corresponding Fisher’s exact test or Kruskal-Wallis rank sum test, with q-values representing false discovery rate correction for multiple testing; p-values smaller than 0.05 were considered significant.

Score distributions of corresponding smell tests in each subject group were illustrated using Cummings estimation plots^41^. The raw UPSIT and SST-ID test scores were also normalized into percentiles based on age and sex, where hyposmia was defined by SST-ID percentile ≤10%^42^ or UPSIT percentile ≤15%^10^. Discrimination performances of these subtests were compared using AUC values with bootstrap estimated 95% CI^43^, in order to distinguish diagnostic groups. Table 2 and Supplementary Table 1 also report optimal thresholds and their associated sensitivity and specificity that correspond to the maximum Youden indices^44^.

### Machine learning workflow of developing and validating an abbreviated smell test

Figure 1 illustrates the machine learning workflow. Data of the Ottawa Trial and baseline data of DeNoPa were used as discovery cohorts, and baseline data of PROBE and follow-up data of DeNoPa were used for (semi-)independent validation. For internal validation, 10-fold cross validation was used: for each smell test, the discovery dataset was randomly partitioned into 10 parts, where the case-control ratio was maintained in each part. In each fold, 9/10 parts were used as the development set and the remaining one part was used for internal validation. This procedure was repeated 10 times, and results were shown either in distribution or average across 10 folds.

Using the corresponding discovery cohorts with 10-fold cross validation, individual scents in SST-ID and UPSIT were ranked separately based on their AUC values in differentiating groups. To control over-fitting, SST-ID and UPSIT scent rankings from this study were compared with eight external rankings^13–20^, four for each test, and two final lists were generated by averaging internal and external rankings. Eleven scents are shared by both smell tests, and therefore, an additional “Shared” ranking was constructed using their respective positions in the averaged SST-ID and UPSIT rankings.

For each scent ranking, beginning with the highest-ranked odorant, subsets were constructed by adding one scent at a time in descending ranking order. A total of 95 and 220 distinct SST-ID and UPSIT subsets with various numbers of scents were compared, using their AUC values in distinguishing PD/DLB from HC, to develop the best-performing simplified tests, including one that unified both smell tests. The resulted abbreviated smell tests were also validated using (semi-)independent datasets.

### Exploring observed differences in scent performance

Percentages of correct scent identification within each subject group were calculated. These percentages were further compared to examine the relationship of scent identification with sex and with age. Within each cohort, participants’ ages were segregated into four bins with similar sample size. For each scent, the proportion of correct identification was calculated for each bin, and spline smoothing was then used to represent the relationship between the proportions and age.

For the IRT analysis, ICCs^22^ for each scent within PD/DLB and HC groups from baseline DeNoPa and Ottawa Trial were used to analyze scent performances and the influence of distractors. The version of ICCs used in this study differed from traditional parametric ICCs in two aspects: 1) the x-axis was the score percentage rank in [0,100], not the latent trait on the whole real line; and 2) ICCs represented spline smoothing lines that fit response data, rather than being fitted to any pre-defined parametric model^22^.

Analyses were performed using ‘R’ (version 4.3.1) with packages: ‘pROC’^43^, ‘dabestr’^45^, ‘TestGardener’^46^, and ‘ggplot2’^47^.

## Supplementary information

Supplementary Information

## Data Availability

This study used three pre-existing, de-identified data sets (DeNoPa, Ottawa Trial, PROBE). We further de-identified these data and made them publicly available on Zenodo (DOI: 10.5281/zenodo.13323913), which limited the reproducibility of certain results involving subjects’ ages and disease duration. If needed, please contact the authors of the original datasets for access to the complete original data.

## Code availability

The code for data analyses and figures is publicly accessible on GitHub https://github.com/JuanLiOHRI/Abbreviated-Smell-Test-PD, and deposited on Zenodo under https://zenodo.org/records/14606736.
