# Supplementary material for: Development of a simplified smell test to identify Parkinson’s disease using multiple cohorts, machine learning and item response theory
Source: NPJ Parkinsons Dis. 2025 Apr 23;11:85. doi: 10.1038/s41531-025-00904-5 (PMC12019603; doi:10.1038/s41531-025-00904-5)
Supplement: Supplementary file 1 — Supplementary Information [file 41531_2025_904_MOESM1_ESM.pdf]

SUPPLEMENTARY APPENDIX

Development of a Simplified Smell Test to Identify Parkinson's Disease Using Multiple Cohorts, Machine Learning and Item Response Theory

Juan Li[1,2,3,4*], Kelsey Grimes[1,2,4], Joseph Saade[1], Julianna J. Tomlinson[1,3,4,5], Tiago A. Mestre[1,2,3,6,7], Sebastian Schade[8], Sandrina Weber[9], Mohammed Dakna[9], Tamara Wicke[8], Elisabeth Lang[8], Claudia Trenkwalder[8,9], Natalina Salmaso[4,10], Andrew Frank[3,11], Tim Ramsay[2,12,13], Douglas Manuel[2,6,13], aSCENT-PD Investigators[4#], Brit Mollenhauer[4,8,9,14*], Michael G. Schlossmacher[1,3,4,5,6,7*]

aSCENT-PD INVESTIGATORS

Ben Arenkiel[4,15], Zhandong Liu[4,15], Brit Mollenhauer[4,8,9,14*], Josef Penninger[4,16], Max Rousseaux[4,5], Armen Saghatelyan[4,5], Natalina Salmaso[4,10], Michael G. Schlossmacher[1,3,4,5,6,7*], Christine Stadelmann[4,17], Julianna J. Tomlinson[1,3,4,5], John M. Woulfe[1,4]

[15] Baylor College of Medicine, Houston, TX, United States

[16] The University of British Columbia, Vancouver, BC, Canada

[17] Institute for Neuropathology, University Medical Center Goettingen, Goettingen, Germany

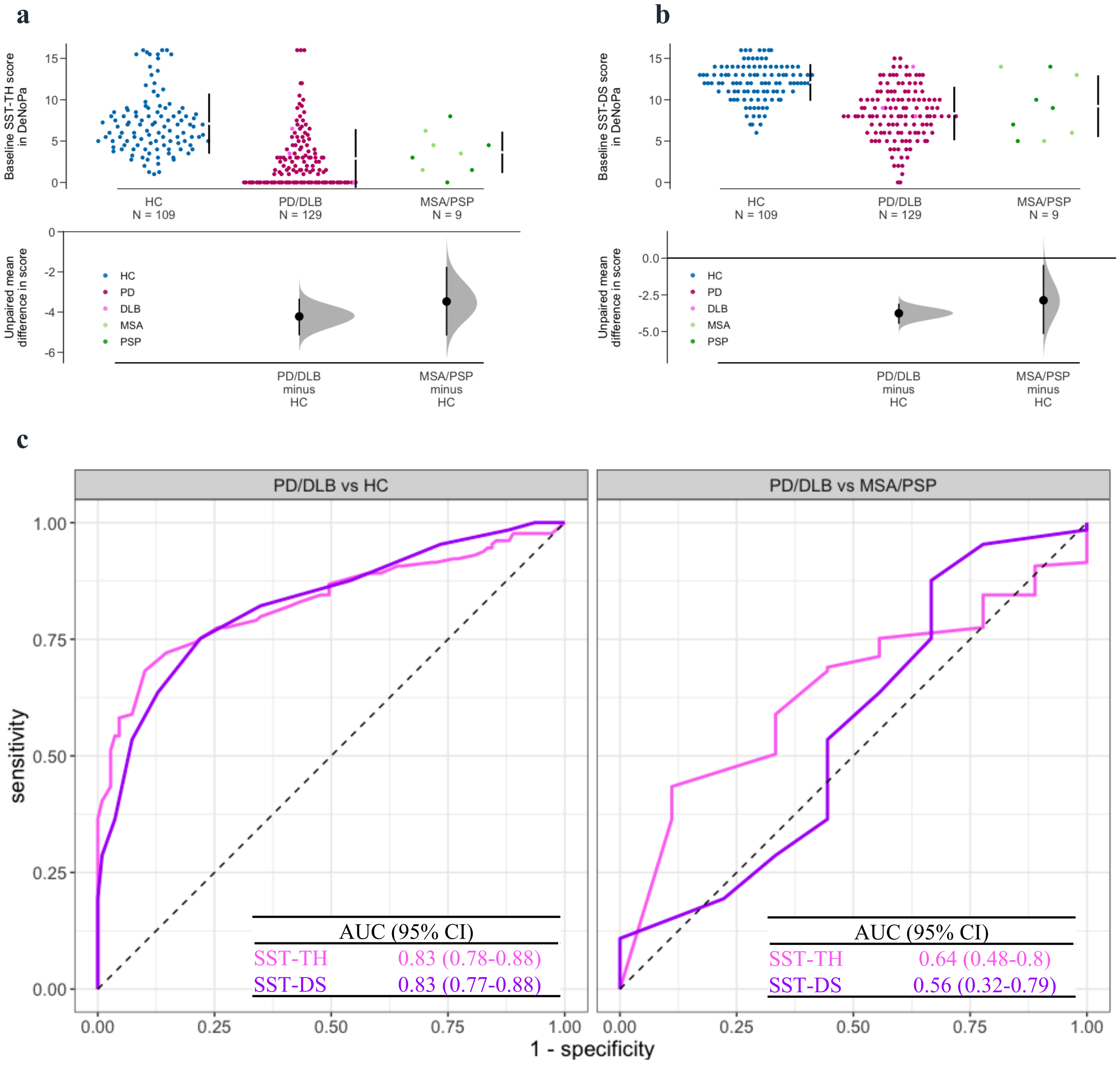

Supplementary Figure 1: Distribution of Sniffin' Sticks Threshold (SST-TH) and Discrimination (SST-DS) scores for each subject group in the DeNoPa Study at baseline and their ROC curves for group classification.

The Cummings estimation plots (a, b) were used to illustrate and compare smell test score distributions in each group: (a) SST-TH, (b) SST-DS. Each data point in the upper panels represents the score of one participant, and colors represent different groups and diagnosis as shown in the legend. The vertical lines in the upper panels represent the conventional mean ± standard deviation error bars. The lower panels show the mean group difference (the effect size) and its 95% confidence interval (CI) estimated by bias-corrected and accelerated bootstrap, using HC as the reference group. Panel (c) shows the ROC curves of each smell test (indicated by color) to distinguish PD/DLB versus HC (left) and PD versus OND (right). HC = healthy control. PD = Parkinson disease. DLB = dementia with Lewy bodies. MSA = multiple system atrophy. PSP = progressive supranuclear palsy. ROC = receiver operating characteristic. AUC = area under the ROC curve.

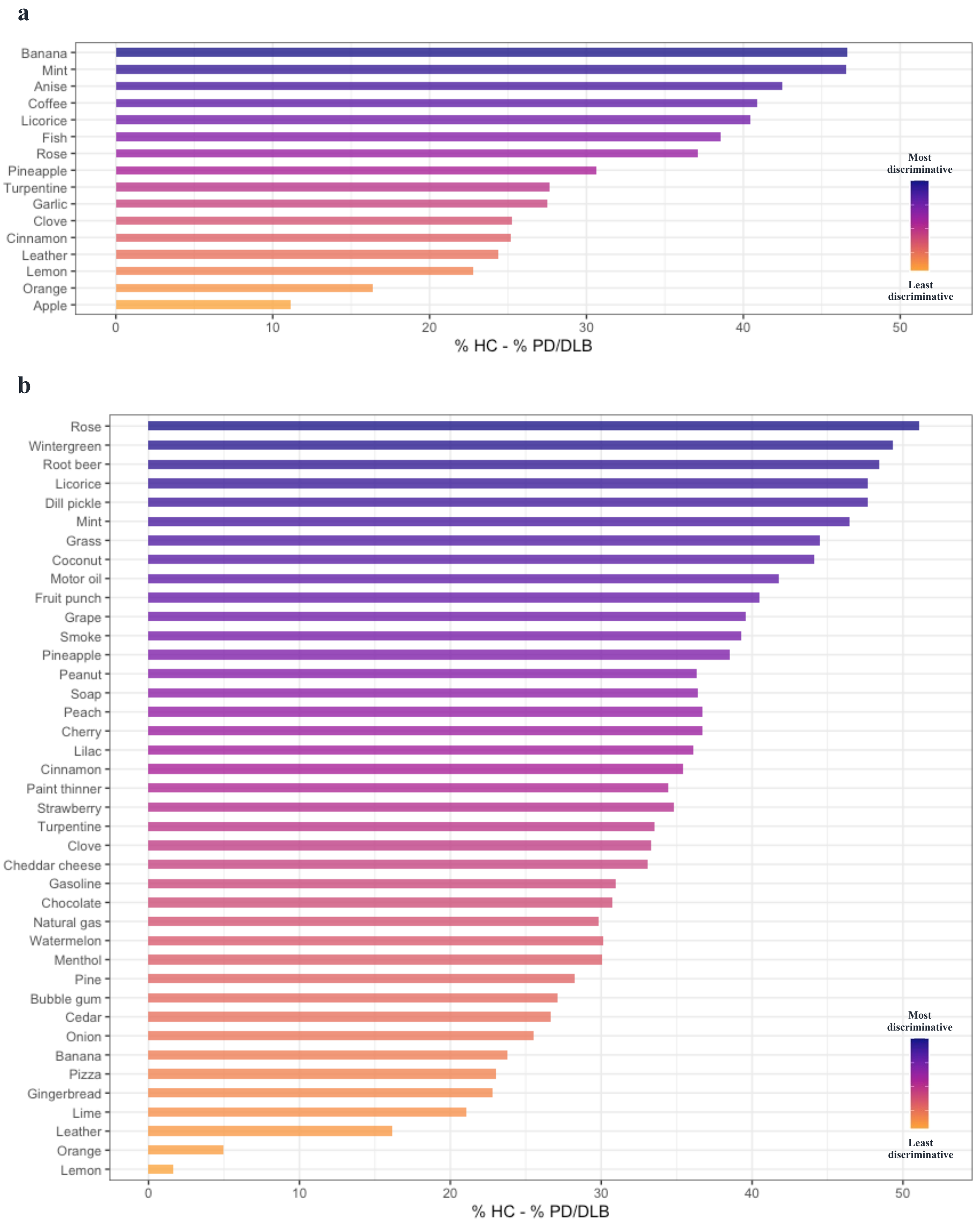

Supplementary Figure 2: Percentage differences in correct scent identification between HC and PD/DLB groups (% HC - % PD/DLB) in the DeNoPa (a) and Ottawa Trial (b) cohorts.
The scents are ordered in descending orders of their mean single-scent AUC value (top to bottom; see **Figure 3** (a) and (c)); the color of each scent changes gradually from the most discriminative to the least discriminative odorant, as indicated by the legend.

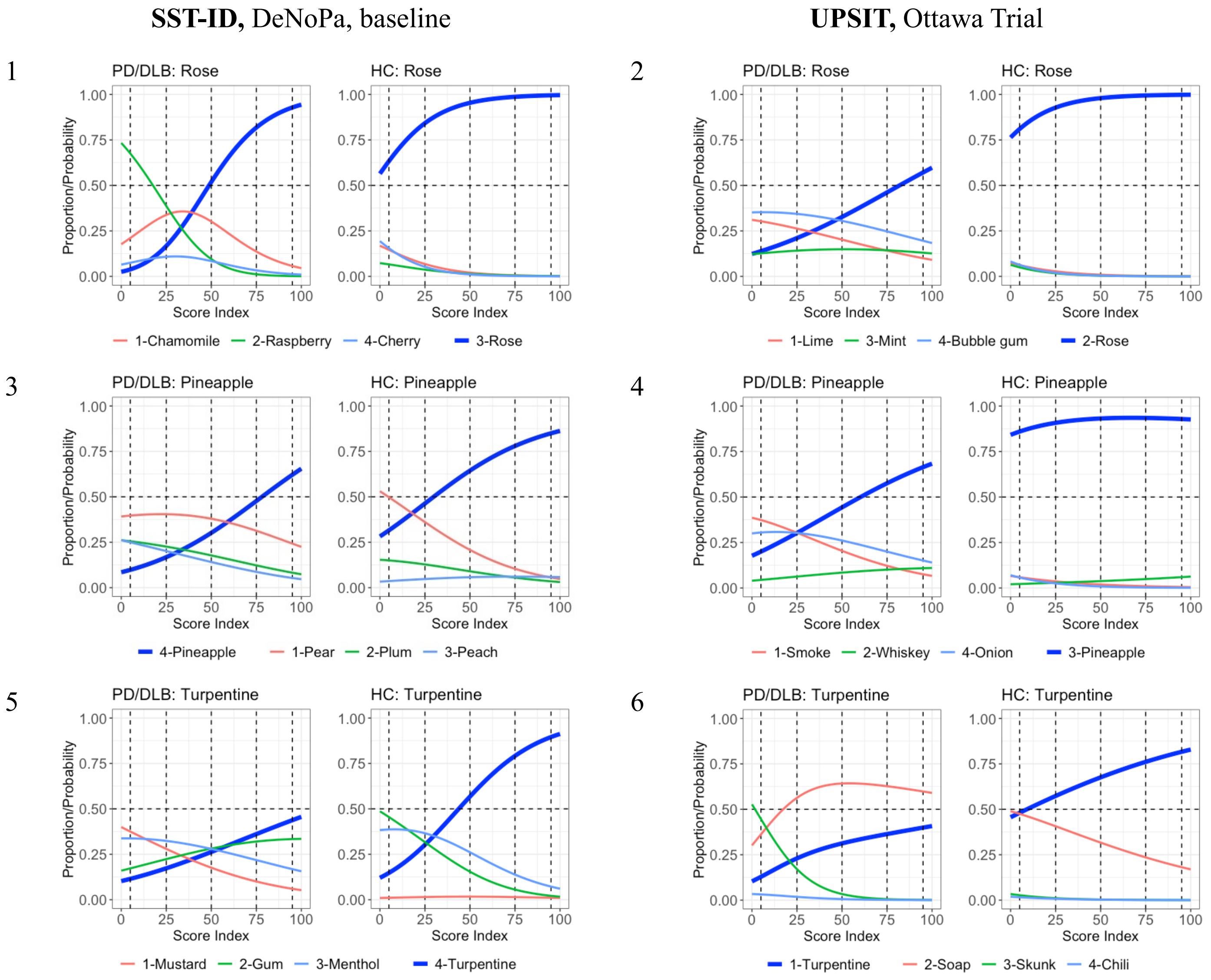

Supplementary Figure 3: Influence of distractors in the multiple-choice smell tests: The remaining six scents shared by UPSIT and SST-ID assays (1/2).

Panels with odd numbers show the Item Characteristic Curves (ICCs) of six SST-ID scents, and panels with even numbers show the ICCs of the corresponding UPSIT scents. In each figure, the left panels are the ICCs using data of the PD/DLB patients, and the right panels are corresponding to healthy controls. The x-axis is transformed score indices in [0,100] (percentage rank of the respective scores) within the corresponding group. The y-axis is the probability of choosing each option at a particular score index. The correct option of each item is highlighted using the thicker blue curves. Numbers in the color legends are the option indices. The horizontal dashed lines represent 50% probability. The vertical dashed lines represent five quantiles (5%, 25%, 50%, 75%, and 95%).

SST-ID, DeNoPa, baseline **UPSIT,** Ottawa Trial

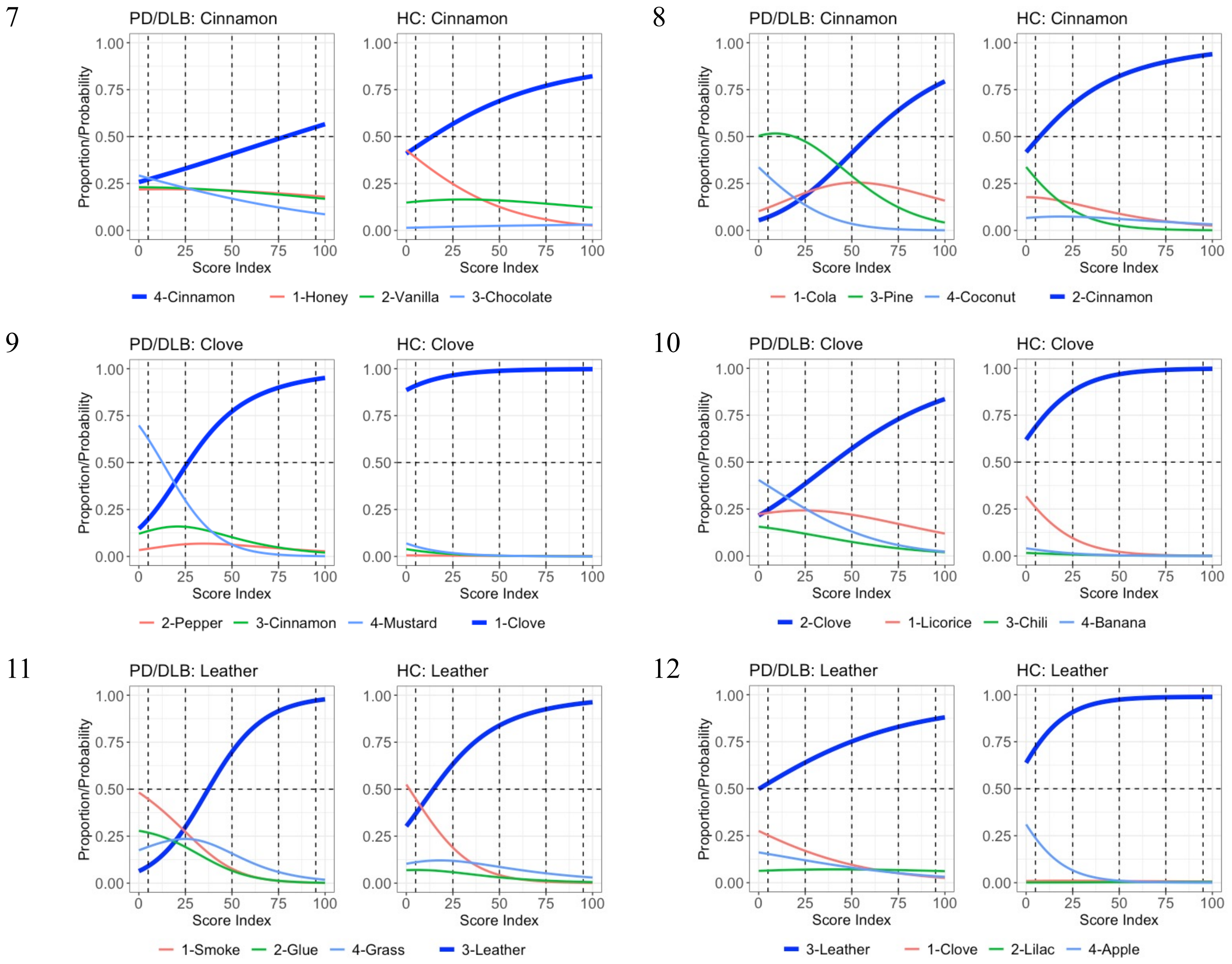

Supplementary Figure 3: Influence of distractors in the multiple-choice smell tests: The remaining six scents shared by UPSIT and SST-ID assays (2/2).

Panels with odd numbers show the Item Characteristic Curves (ICCs) of six SST-ID scents, and panels with even numbers show the ICCs of the corresponding UPSIT scents. In each figure, the left panels are the ICCs using data of the PD/DLB patients, and the right panels are corresponding to healthy controls. The x-axis is transformed score indices in [0,100] (percentage rank of the respective scores) within the corresponding group. The y-axis is the probability of choosing each option at a particular score index. The correct option of each item is highlighted using the thicker blue curves. Numbers in the color legends are the option indices. The horizontal dashed lines represent 50% probability. The vertical dashed lines represent five quantiles (5%, 25%, 50%, 75%, and 95%).

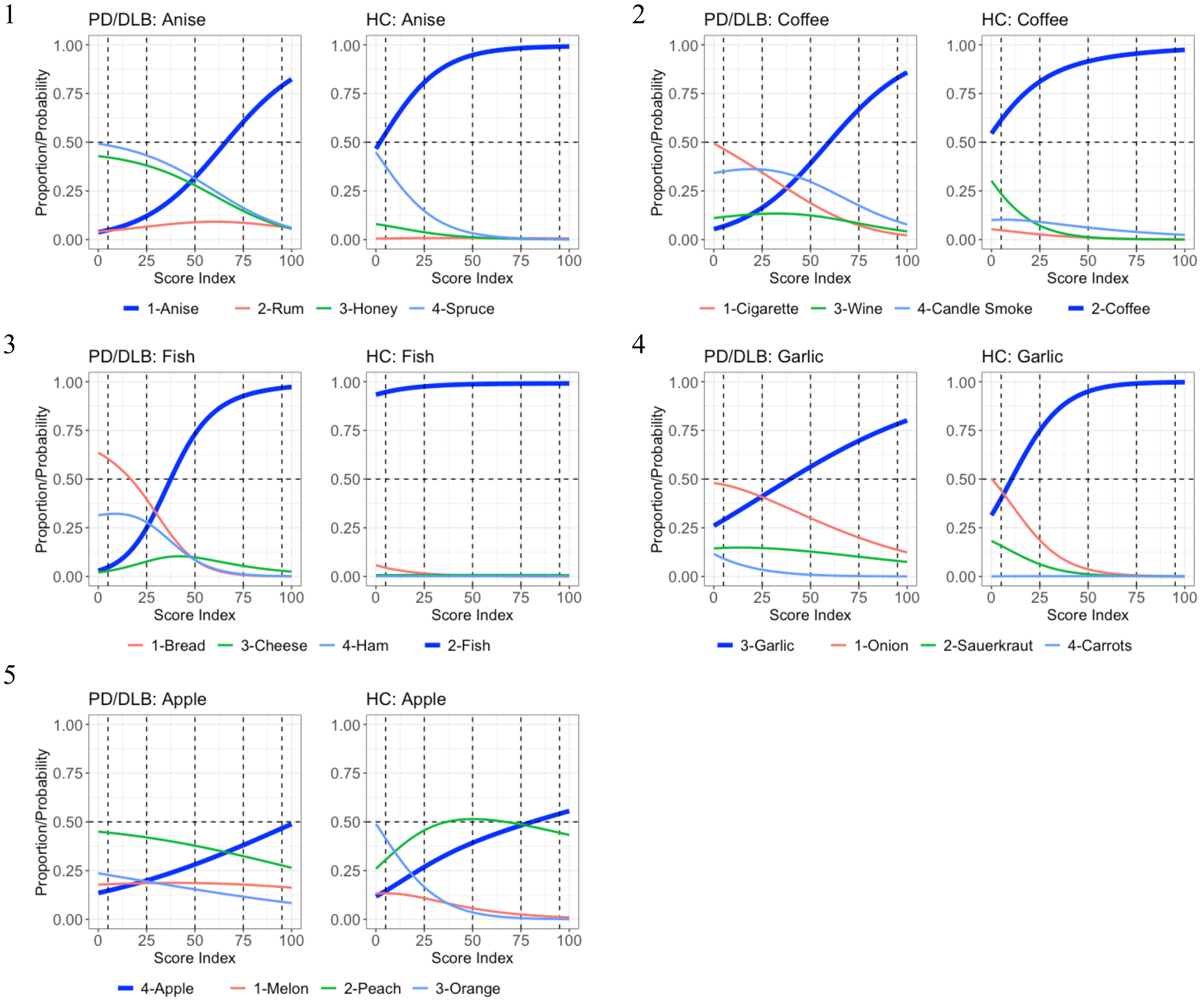

Supplementary Figure 4: Item Characteristic Curves (ICCs) of the remaining SST-ID scents using baseline data from the DeNoPa Cohort.

In each panel, the left panels are the ICCs using data of the PD/DLB patients, and the right panels are corresponding to healthy controls. The x-axis is transformed score indices in [0,100] (percentage rank of the SST-ID score) within PD and HC group, respectively. The y-axis is the probability of choosing each option at a particular score index. The correct option of each item is highlighted using the thicker blue curves. Numbers in the color legends are the option indices. The horizontal dashed lines represent 50% probability. The vertical dashed lines represent five quantiles (5%, 25%, 50%, 75%, and 95%).

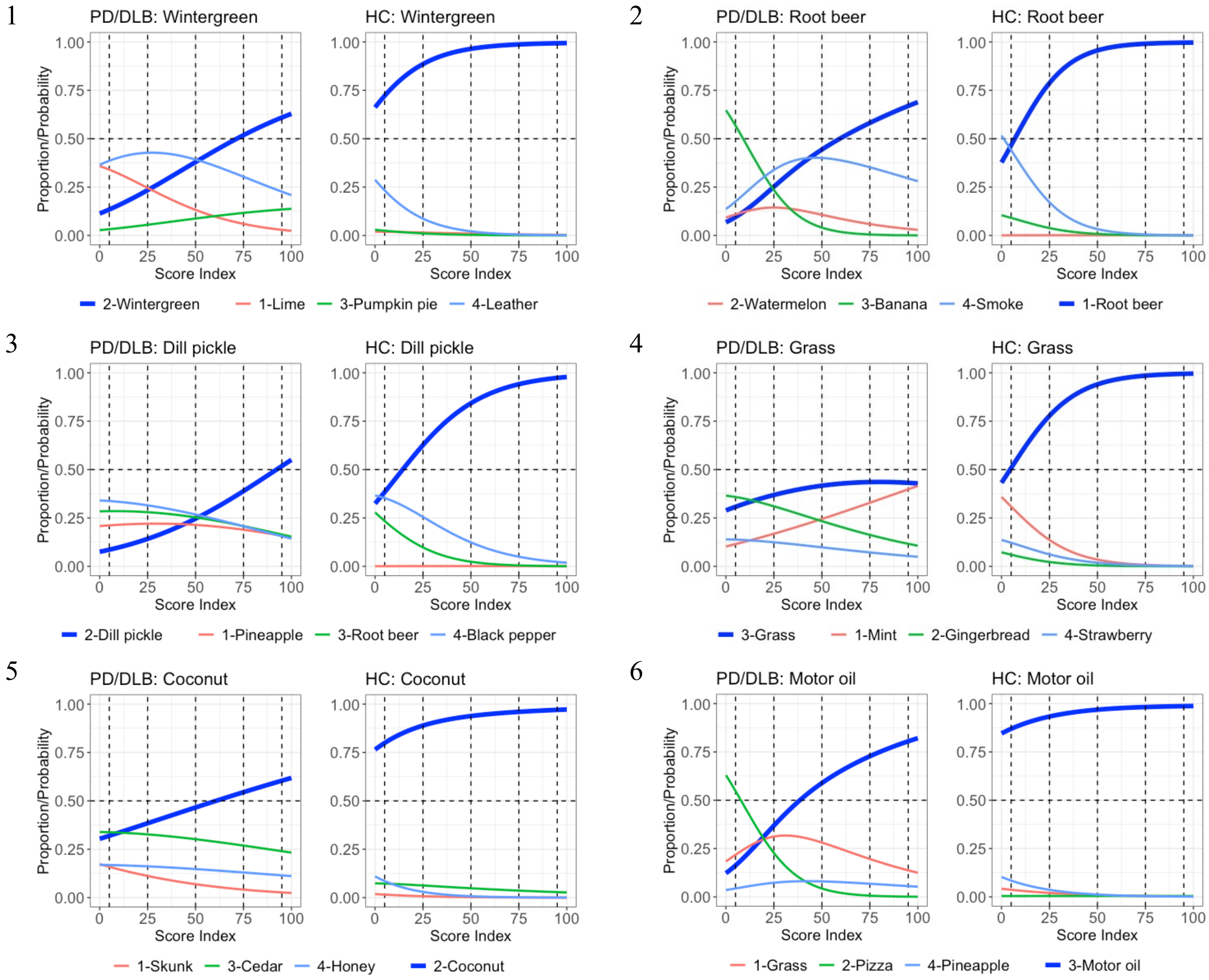

Supplementary Figure 5: Item Characteristic Curves (ICCs) of the remaining UPSIT scents using the Ottawa Trial cohort (1/5).

In each panel, the left panels are the ICCs using data of the PD/DLB patients, and the right panels are corresponding to healthy controls. The x-axis is transformed score indices in [0,100] (percentage rank of the UPSIT score) within PD and HC group, respectively. The y-axis is the probability of choosing each option at a particular score index. The correct option of each item is highlighted using the thicker blue curves. Numbers in the color legends are the option indices. The horizontal dashed lines represent 50% probability. The vertical dashed lines represent five quantiles (5%, 25%, 50%, 75%, and 95%).

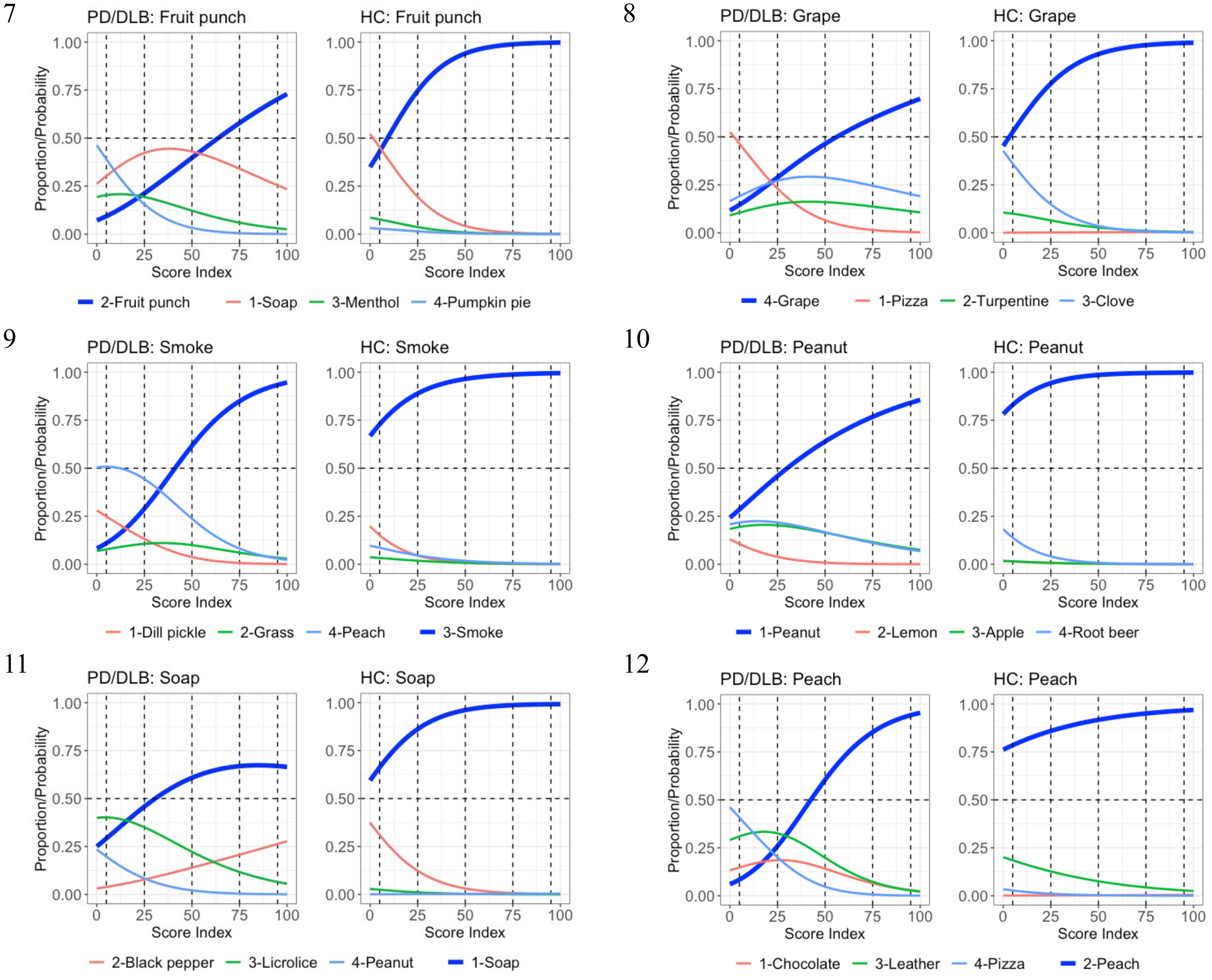

Supplementary Figure 5: Item Characteristic Curves (ICCs) of the remaining UPSIT scents using the Ottawa Trial cohort (2/5).

In each panel, the left panels are the ICCs using data of the PD/DLB patients, and the right panels are corresponding to healthy controls. The x-axis is transformed score indices in [0,100] (percentage rank of the UPSIT score) within PD and HC group, respectively. The y-axis is the probability of choosing each option at a particular score index. The correct option of each item is highlighted using the thicker blue curves. Numbers in the color legends are the option indices. The horizontal dashed lines represent 50% probability. The vertical dashed lines represent five quantiles (5%, 25%, 50%, 75%, and 95%).

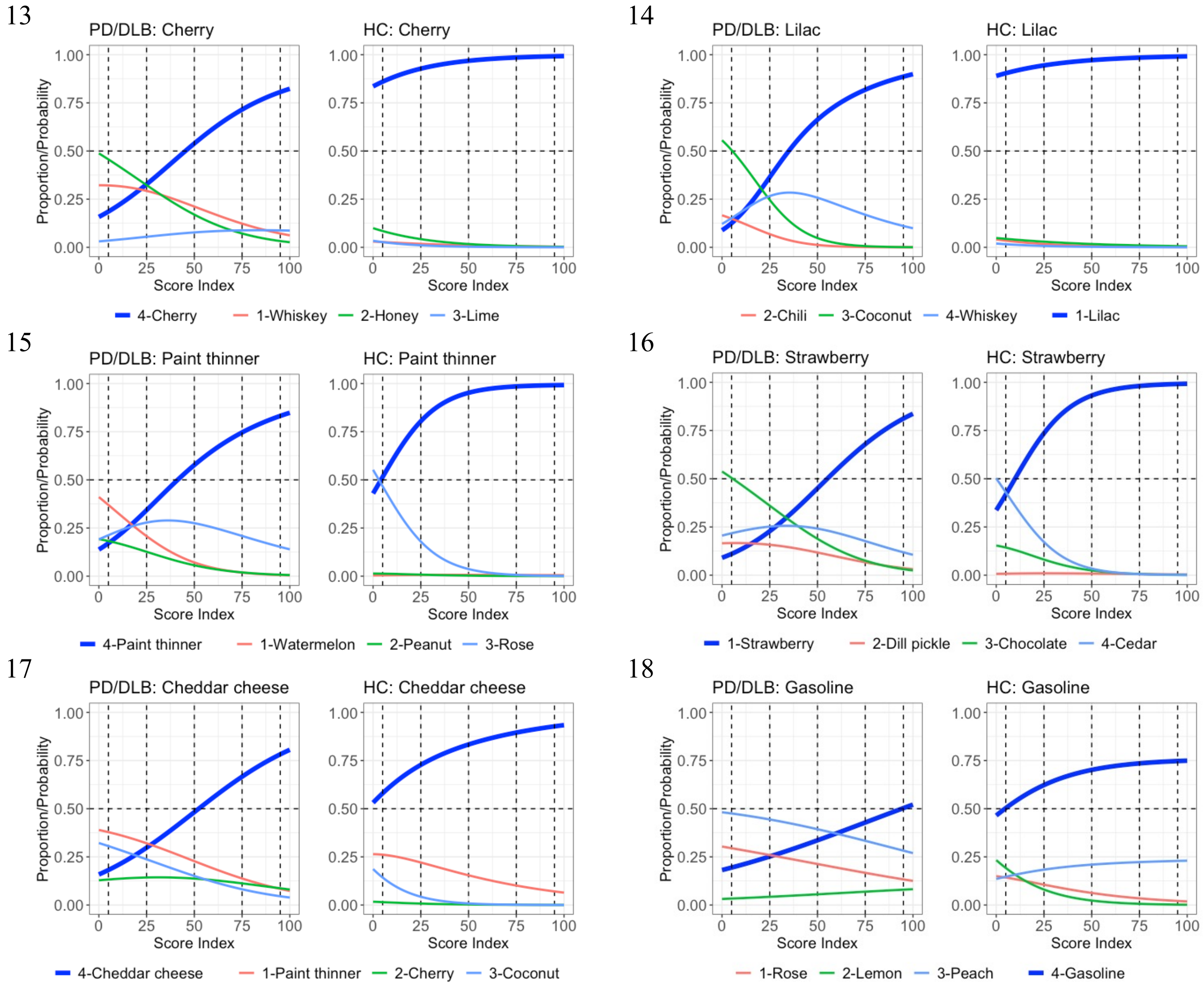

Supplementary Figure 5: Item Characteristic Curves (ICCs) of the remaining UPSIT scents using the Ottawa Trial cohort (3/5).

In each panel, the left panels are the ICCs using data of the PD/DLB patients, and the right panels are corresponding to healthy controls. The x-axis is transformed score indices in [0,100] (percentage rank of the UPSIT score) within PD and HC group, respectively. The y-axis is the probability of choosing each option at a particular score index. The correct option of each item is highlighted using the thicker blue curves. Numbers in the color legends are the option indices. The horizontal dashed lines represent 50% probability. The vertical dashed lines represent five quantiles (5%, 25%, 50%, 75%, and 95%).

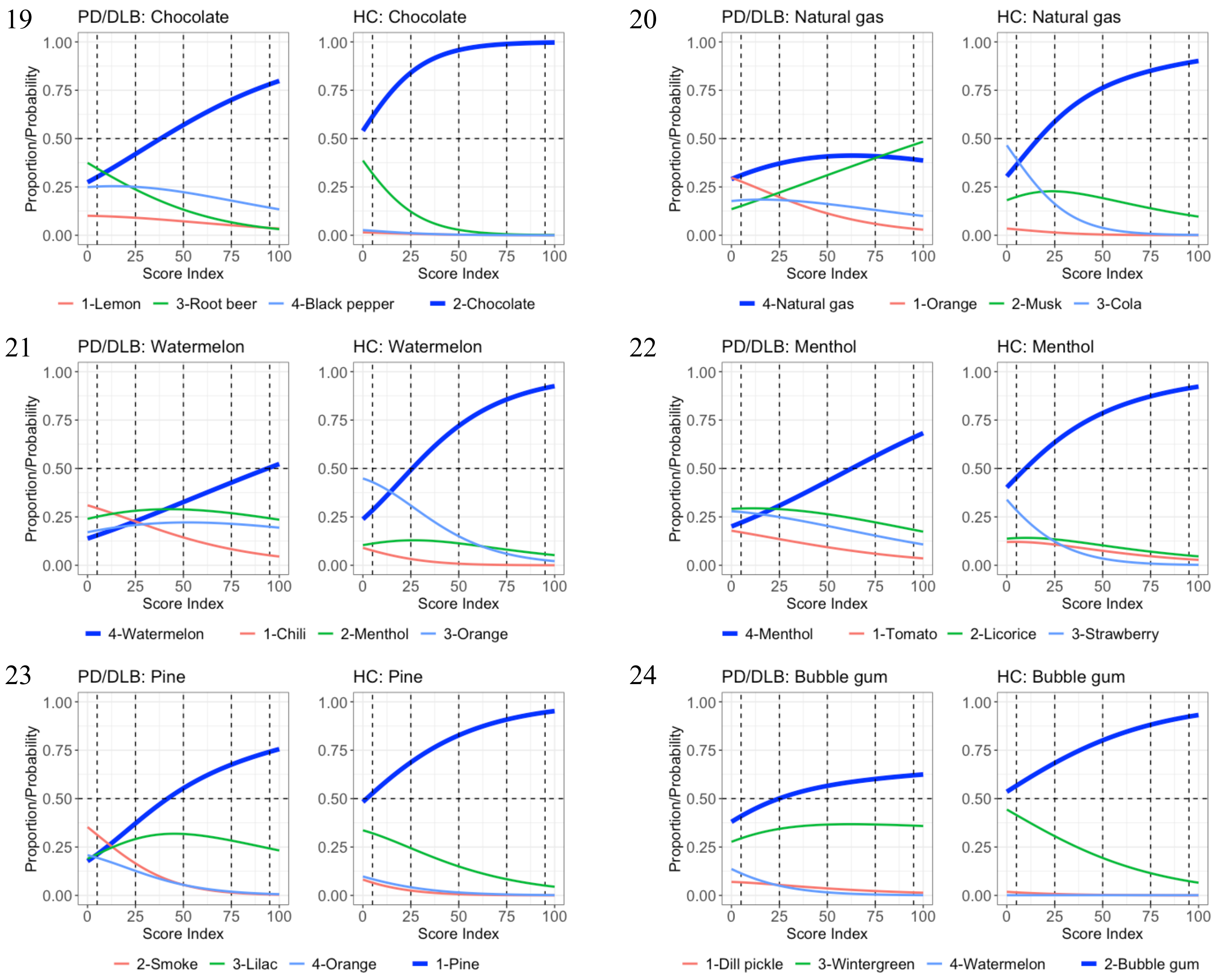

Supplementary Figure 5: Item Characteristic Curves (ICCs) of the remaining UPSIT scents using the Ottawa Trial cohort (4/5).

In each panel, the left panels are the ICCs using data of the PD/DLB patients, and the right panels are corresponding to healthy controls. The x-axis is transformed score indices in [0,100] (percentage rank of the UPSIT score) within PD and HC group, respectively. The y-axis is the probability of choosing each option at a particular score index. The correct option of each item is highlighted using the thicker blue curves. Numbers in the color legends are the option indices. The horizontal dashed lines represent 50% probability. The vertical dashed lines represent five quantiles (5%, 25%, 50%, 75%, and 95%).

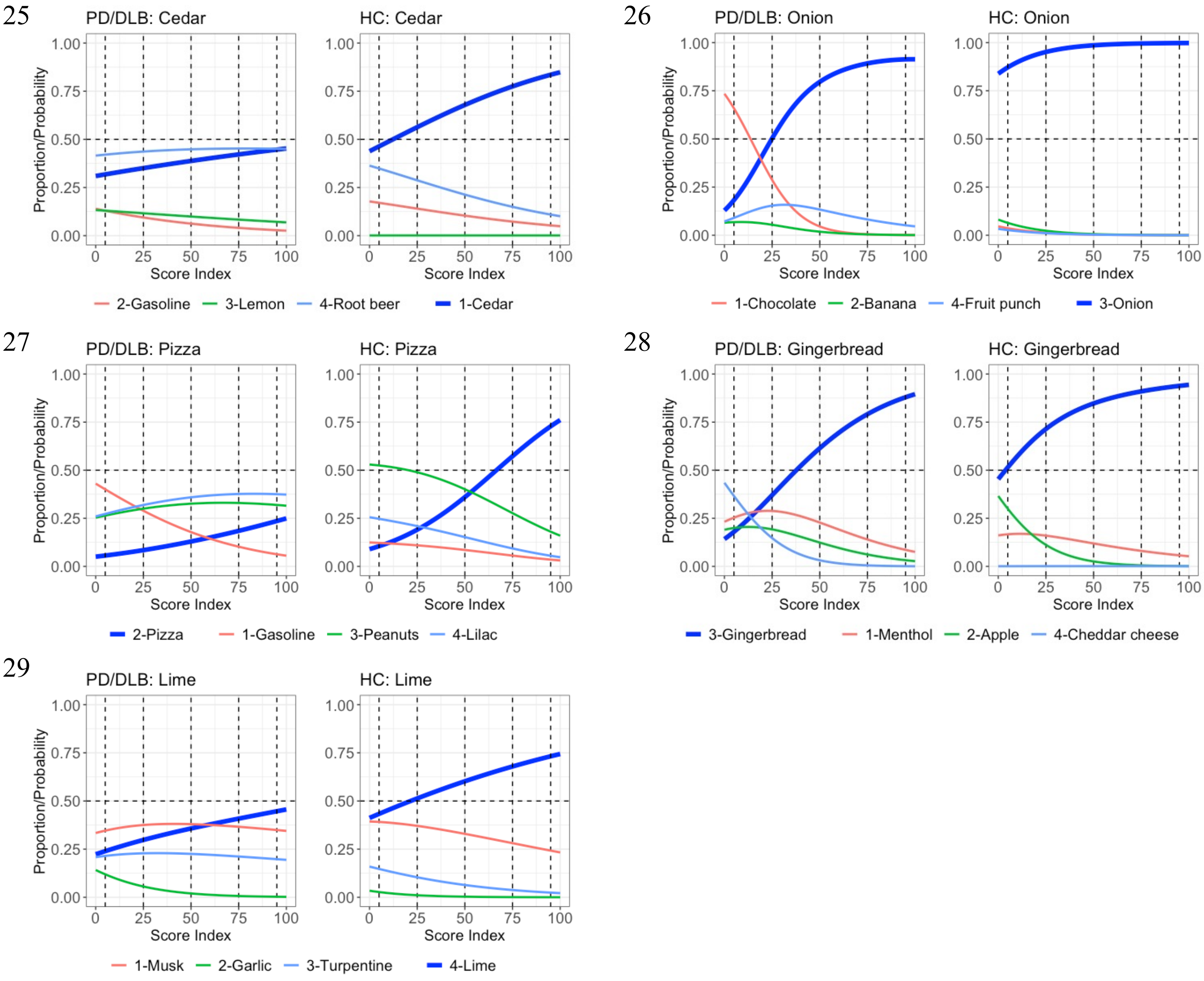

Supplementary Figure 5: Item Characteristic Curves (ICCs) of the remaining UPSIT scents using the Ottawa Trial cohort (5/5).

In each panel, the left panels are the ICCs using data of the PD/DLB patients, and the right panels are corresponding to healthy controls. The x-axis is transformed score indices in [0,100] (percentage rank of the UPSIT score) within PD and HC group, respectively. The y-axis is the probability of choosing each option at a particular score index. The correct option of each item is highlighted using the thicker blue curves. Numbers in the color legends are the option indices. The horizontal dashed lines represent 50% probability. The vertical dashed lines represent five quantiles (5%, 25%, 50%, 75%, and 95%).

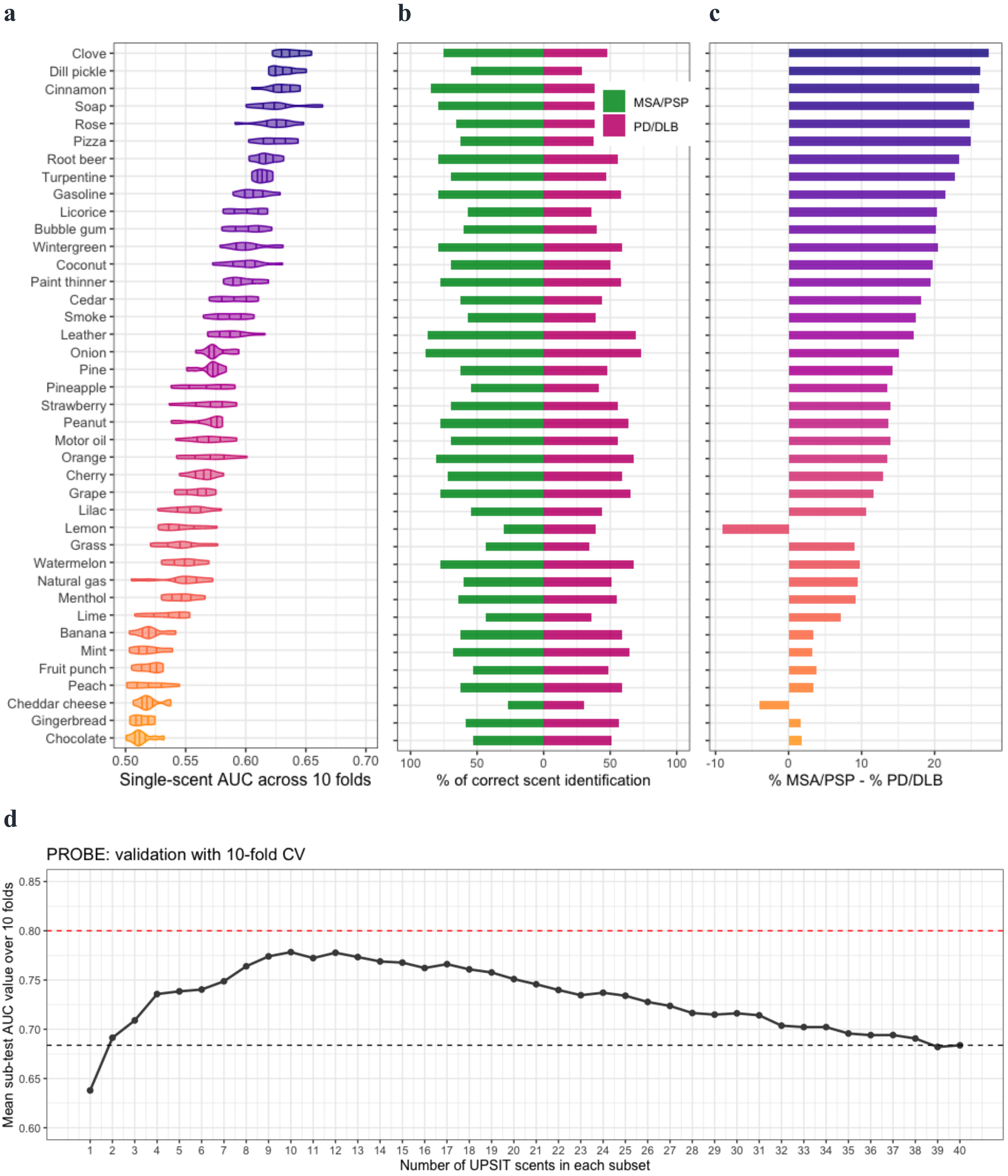

Supplementary Figure 6: Range of performances for UPSIT-based odorants in differentiating PD/DLB from MSA/PSP subjects in the PROBE cohort and AUC values for numerical subsets of scents in group classification.

Panel (a) illustrates distribution of AUC values of each scent across 10-fold cross-validation using violin plots, with 25%, 50%, and 75% quantile lines. The scents are ordered in descending order of their mean single-scent AUC value. The color of each scent changes gradually from the most to the least discriminative odorant, as indicated by the legend. Panel (b) shows the percentage of subjects correctly identifying each scent within the MSA/PSP and PD/DLB groups, and panel (c) shows the percentage differences between the two groups. Scents in panels (b) and (c) follow the same rank order as in panel (a). In panel (d), the x-axis is the number of scents included for each subset, with individual points representing average AUC values for the validation set across ten CV folds gathered in PROBE. The black horizontal, dashed line indicates the corresponding AUC values of the whole test (= 40 scents). The red horizontal, dashed line indicates AUC = 0.8 as a predetermined reference line.

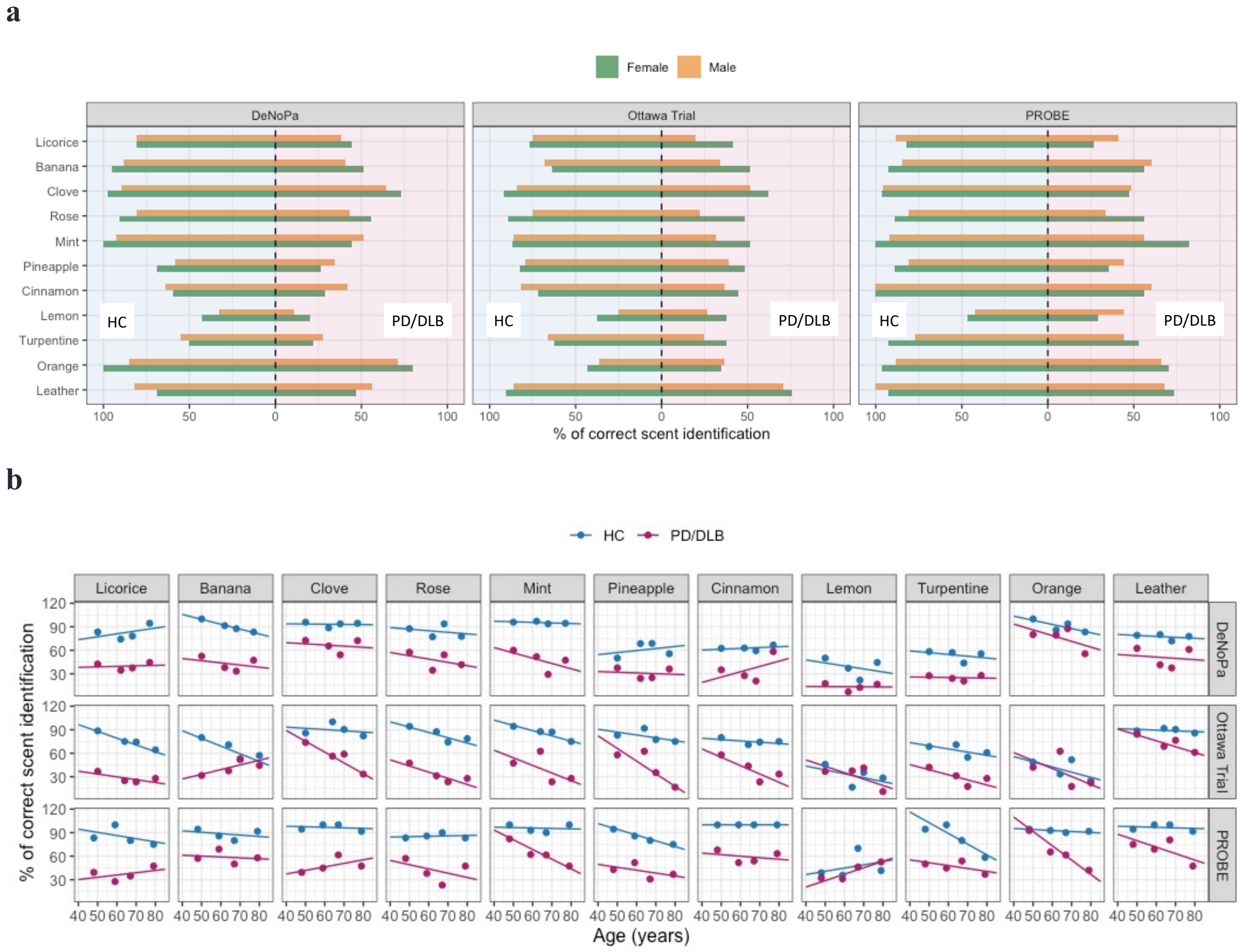

Supplementary Figure 7: Relationships between scent identification performance, diagnosis, sex and age.

Panel (a) shows the percentage of persons that correctly identified each scent within the healthy control (HC) group (indicated by light blue region) and the PD/DLB group (indicated by pink region) in the corresponding cohorts, separated by sex (bar color). Panels in (b) show the relationship between age (x-axis), diagnostic group (HC in blue; vs PD/DLB in red) and the percentage of correctly identified scents (y-axis) for each odorant tested (columns) within each cohort (row), as indicated on the right.

SUPPLEMENTARY TABLES

Supplementary Table 1: Discriminative performances of complete smell tests for baseline visits in three cohorts

Cohort	Test	AUC (95% CI)	Threshold	Sensitivity	Specificity
**PD/DLB vs HC**					
	SST-ID	0.89 (0.85-0.93)	≤ 10	0.88	0.81
DeNoPa	SST-TH	0.83 (0.78-0.88)	≤ 3.12	0.68	0.9
	SST-DS	0.83 (0.77-0.88)	≤ 10	0.75	0.78
Ottawa trial	UPSIT	0.92 (0.88-0.96)	≤ 28	0.96	0.77
PROBE	UPSIT	0.93 (0.89-0.97)	≤ 29	0.84	0.89
**PD/DLB vs MSA/PSP**					
	SST-ID	0.8 (0.69-0.91)	≤ 6	0.48	1
DeNoPa	SST-TH	0.64 (0.48-0.8)	≤ 1.38	0.43	0.89
	SST-DS	0.56 (0.32-0.79)	≤ 12	0.88	0.33
Ottawa trial	UPSIT	0.92 (0.85-0.99)	≤ 25	0.81	1
PROBE	UPSIT	0.69 (0.6-0.78)	≤ 26	0.76	0.58
**PD/DLB vs Other (HC and MSA/PSP)**					
	SST-ID	0.89 (0.84-0.93)	≤ 10	0.88	0.78
DeNoPa	SST-TH	0.81 (0.76-0.87)	≤ 3.12	0.68	0.87
	SST-DS	0.8 (0.75-0.86)	≤ 10	0.75	0.75
Ottawa trial	UPSIT	0.92 (0.88-0.96)	≤ 28	0.96	0.76
PROBE	UPSIT	0.81 (0.75-0.87)	≤ 26	0.76	0.78

DeNoPa = *De Novo* Parkinson Study. PROBE = Prognostic Biomarkers in Parkinson Disease. HC = healthy control. PD = Parkinson disease. DLB = dementia with Lewy bodies. MSA = multiple system atrophy. PSP = progressive supranuclear palsy. AUC = area under the ROC curve. CI = confidence interval. SST-ID = Sniffin' Sticks Identification test (score range: 0-16). SST-TH = Sniffin' Sticks Threshold test (score range: 0-16). SST-DS = Sniffin' Sticks Discrimination test (score range: 0-16). UPSIT = University of Pennsylvania Smell Identification Test (score range: 0-40).

Supplementary Table 2: Comparison of cohorts and methods used in this study and eight published studies

	UPSIT					SST-ID				
	This study	**Bohnen *et al.***	**Hawkes *et al.***	**Joseph *et al.*[8]**	**Morley *et al.***	***This study***	**Boesveldt *et al.***	**Casjens *et al.***	**Lo *et al.*[9]**	**Mahlknecht *et al.***
Study cohorts										
**Discovery**										
Cohort name/location	Ottawa Trial	University of Pittsburgh	Ipswich	PREDICT-PD	Michael J. Crescenz VA Medical Center in Philadelphia and the University of Pennsylvania	DeNoPa	VUMC; LUMC	ParkCHIP	Oxford Discovery	Innsbruck and Bruneck
PD	**70 (37.2)**[1,6]	**27 (50)**[1]	**96 (50)**[1]	**40 (4.3)**[1]	**314 (50)**[1]	**129 (54.2)**[1,7]	**404 (72.9)**[1]	**148 (50)**[1]	**890 (74)**[1]	**134 (28.5)**[1]
Male sex	41 (59)[1]	20 (74.1)[1]	49 (51)[1]	30 (75)[1]	261 (83)[1]	84 (65)[1]	253 (62.6)[1]	78 (52.7)[1]	569 (64)[1]	84 (62.7)[1]
Age in years	68 (60, 74)[2]	60 (11.1)[3]	57 (27-81)[4]	63.8 (9.6)[3]	67.4 (10.0)[3]	66 (58, 72)[2]	61.5 (40-90)[4]	67 (14)[2]	66.5 (9.6)[3]	68.0 (8.8)[3]
PD duration in years	7 (3, 11)[2]	2.5 (2.7)[3]	unknown	unknown	unknown	1.2 (0.75, 2)[2]	0-44[5]	n.a.	1.2 (0.9)[3]	6.2 (4.8)[3]
HC	**118 (62.8)**[1]	**27 (50)**[1]	**96 (50)**[1]	**891 (95.7)**[1]	**314 (50)**[1]	**109 (45.8)**[1]	**150 (27.1)**[1]	**148 (50)**[1]	**313 (26)**[1]	**336 (71.5)**[1]
Male	44 (37)[1]	20 (74.1)[1]	39 (40.6)[1]	343 (38.5)[1]	261 (83)[1]	67 (61)[1]	87 (58)[1]	81 (54.7)[1]	165 (53)[1]	156 (46.4)[1]
Age in years	68 (58, 73)[2]	60 (7)[3]	41.7 (18-78)[4]	67.3 (4.8)[3]	67.4 (10.0)[3]	65 (60, 70)[2]	59.2 (45-78)[4]	62 (16)[2]	64.4 (9.8)[3]	68.8 (8.3)[3]
**(Semi-)External Validation**	PROBE: PD = 102, HC = 54	n.a.	n.a.	PREDICT-PD: HC = 191, who have completed UPSIT in only year 3.	UCL: PD = 167, HC = 130 Barts: PD = 176, HC = 177	DeNoPa at 48 months: PD=114, HC=101 DeNoPa at 72 months: PD=91, HC=93	n.a.	n.a.	Tracking cohort: 452	VUMC; LUMC: PD = 400, HC = 150 Vienna: PD = 112, HC = 120
Methods										
Internal validation	10-fold cross-validation	n.a.	n.a.	n.a.	n.a.	10-fold cross-validation	n.a.	10-fold cross-validation	data balance with leave-one-out cross-validation (LOOCV)	n.a.

Ranking scents	AUC values of each question in distinguishing PD/DLB from HC	Difference of percentages responding correctly in each group (%HC - %PD)	Difference of percentages responding correctly in each group (%HC - %PD)	Difference of percentages responding correctly in each group (%HC - %PD)	40 scents were ranked using 5 methods and only the top-12 of each ranking were reported: 1) the absolute difference in percentage of PD and control subjects answering incorrectly (Difference), 2) odds ratio, 3) discriminant function analysis (Discriminant),4) logistic regression (Regression), 5) a weighted average combining the first four methods (Combined).	AUC values of each question in distinguishing PD from HC	Difference of percentages responding correctly in each group (%HC - %PD)	Random forest with permutation accuracy	Random forest with predictor importance (Gini diversity index)	L1-regularized logistic regression implementing the least absolute shrinkage and selection operator (the LASSO)

Values are [1]n (%), [2]median (Inter-quartile range (IQR)), [3]mean (standard deviation (SD)), [4]mean (range), [5]range,

[6]Also includes one patient with dementia with Lewy bodies (DLB). [7]Also includes three patients with DLB.

[8]The paper assessed all combinations of 1–7 smells from UPSIT and identified 28 "winning" smell combinations that had highest combined sensitivity and specificity to define hyposmia within the 891 healthy controls in PREDIGT-PD. The ranking below was not directly used to develop the optimal subset of scents.

[9]The discovery cohort was 267 HC with good (normosmia/super-smeller) sense of smell and 721 PD with poor (functional anosmia/hyposmia) sense of smell as defined by age- and sex-specific percentiles: functional anosmia: SST-ID scores ≤ 8; hyposmia: < 10th percentile; super-smeller: > 90th percentile. The outcome was to classify participants with poor vs normal smell, not to classify PD vs HC, nor conversion to PD in the iRBD cohort.

Supplementary Table 3: Ranking of scents shared by SST-ID and UPSIT tests

Rank[1]	Scent	Rank in SST-ID[2]	Rank in UPSIT[2]	Average[3]
1	Licorice	2/16	5/40	0.125
2	Banana	4/16	4/40	0.175
3	Clove	8/16	2/40	0.275
4	Rose	7/16	9/40	0.33125
5	Mint	3/16	21/40	0.35625
6	Pineapple	9/16	8/40	0.38125
7	Cinnamon	10/16	16/40	0.5125
8	Lemon	12/16	23/40	0.6625
9	Turpentine	13/16	27/40	0.74375
10	Orange	15/16	29/40	0.83125
11	Leather	11/16	40/40	0.84375

[1] Smaller number in ranking means better discriminative performance, *e.g.*, rank #1 represents the most discriminative scent.

[2] Based on the corresponding "Average" rankings in **Figure 4**.

[3] For each scent, average = (rank in SST-ID + rank in UPSIT) / 2

SST-ID = Sniffin' Sticks Identification test. UPSIT = University of Pennsylvania Smell Identification Test.

Supplementary Table 4: Relationship between the SST-ID score (DeNoPa) or UPSIT score (Ottawa Trial; PROBE) with age, sex, and diagnostic groups

	**DeNoPa**			**Ottawa Trial**			**PROBE**		
**Variable**	**Beta**	**95% CI[1]**	**p-value**	**Beta**	**95% CI[1]**	**p-value**	**Beta**	**95% CI[1]**	**p-value**
**Age**	-0.05	-0.09, -0.01	**0.022**	-0.22	-0.31, -0.14	**<0.001**	-0.21	-0.31, -0.12	**<0.001**
**Sex**									
Female	—	—		—	—		—	—	
Male	-0.66	-1.4, 0.06	0.073	-2.9	-4.6, -1.2	**<0.001**	-1.9	-3.9, 0.07	0.06
**Group**									
HC	—	—		—	—		—	—	
PD/DLB	-5	-5.7, -4.3	**<0.001**	-13	-14, -11	**<0.001**	-13	-15, -11	**<0.001**
MSA/PSP	-1.4	-3.3, 0.51	0.2	-1.8	-6.5, 2.9	0.5	-6.4	-9.2, -3.7	**<0.001**

[1] CI = Confidence Interval

Supplementary Table 5: Standards for Reporting of Diagnostic Accuracy Studies (STARD) checklist

Section & Topic	No	Item	Reported on page #
**TITLE OR ABSTRACT**			
	**1**	Identification as a study of diagnostic accuracy using at least one measure of accuracy (such as sensitivity, specificity, predictive values, or AUC)	Title (P1)<br>Abstract (P4)
**ABSTRACT**			
	**2**	Structured summary of study design, methods, results, and conclusions (for specific guidance, STARD for Abstracts)	Abstract (P4)
**INTRODUCTION**			
	**3**	Scientific and clinical background, including the intended use and clinical role of the index test	Introduction (P5-6)
	**4**	Study objectives and hypotheses	Introduction (P5-6)
**METHODS**			
Study design	**5**	Whether data collection was planned before the index test and reference standard were performed (prospective study) or after (retrospective study)	Methods: Source of data and participants (P15-17)
Participants	**6**	Eligibility criteria	Methods: Source of data and participants (P15-17)
	**7**	On what basis potentially eligible participants were identified (such as symptoms, results from previous tests, inclusion in registry)	Methods: Source of data and participants (P15-17)
	**8**	Where and when potentially eligible participants were identified (setting, location and dates)	Methods: Source of data and participants (P15-17)
	**9**	Whether participants formed a consecutive, random or convenience series	Methods: Source of data and participants (P15-17)
Test methods	**10a**	Index test, in sufficient detail to allow replication	Methods: Study assessments (P17)
	**10b**	Reference standard, in sufficient detail to allow replication	Methods: Source of data and participants (P15-17)
	**11**	Rationale for choosing the reference standard (if alternatives exist)	Methods: Source of data and participants (P15-17)
	**12a**	Definition of and rationale for test positivity cut-offs or result categories of the index test, distinguishing pre-specified from exploratory	Methods: Data preparation and analysis (P17-18)
	**12b**	Definition of and rationale for test positivity cut-offs or result categories of the reference standard, distinguishing pre-specified from exploratory	Methods: Source of data and participants (P15-17)
	**13a**	Whether clinical information and reference standard results were available to the performers/readers of the index test	Methods: Source of data and participants (P15-17)
	**13b**	Whether clinical information and index test results were available to the assessors of the reference standard	Methods: Source of data and participants (P15-17)
Analysis	**14**	Methods for estimating or comparing measures of diagnostic accuracy	Methods: Data preparation and analysis, Machine learning workflow of developing and validating an abbreviated smell test (P17-19)
	**15**	How indeterminate index test or reference standard results were handled	Methods: Data preparation and analysis, Machine learning workflow of developing and validating an abbreviated smell test (P17-19)
	**16**	How missing data on the index test and reference standard were handled	Methods: Data preparation and analysis (P17-18)
	**17**	Any analyses of variability in diagnostic accuracy, distinguishing pre-specified from exploratory	Methods: Data preparation and analysis, Machine learning workflow of developing and validating an abbreviated smell test, and Exploring observed differences in scent performance (P17-19)
	**18**	Intended sample size and how it was determined	Methods: Source of data and participants (P15-17), secondary use of data
**RESULTS**			
Participants	**19**	Flow of participants, using a diagram	Methods: Source of data and participants (P15-17)<br>Results (P6)
	**20**	Baseline demographic and clinical characteristics of participants	Table 1
	**21a**	Distribution of severity of disease in those with the target condition	Table 1,<br>Methods: Source of data and participants (P15-17)

	**21b**	Distribution of alternative diagnoses in those without the target condition	Table 1
	**22**	Time interval and any clinical interventions between index test and reference standard	Methods: Source of data and participants (P15-17)
Test results	**23**	Cross tabulation of the index test results (or their distribution) by the results of the reference standard	Figures 2, 6<br>Supplementary Figure 1<br>Tables 1, 2<br>Supplementary Table 1
	**24**	Estimates of diagnostic accuracy and their precision (such as 95% confidence intervals)	Figures 2, 6<br>Supplementary Figure 1<br>Tables 1, 2<br>Supplementary Table 1
	**25**	Any adverse events from performing the index test or the reference standard	The smell tests are non-invasive and there was no adverse event from performing them. P13
**DISCUSSION**			
	**26**	Study limitations, including sources of potential bias, statistical uncertainty, and generalisability	Discussion (P14-15)
	**27**	Implications for practice, including the intended use and clinical role of the index test	Discussion (P13-15)
**OTHER INFORMATION**			
	**28**	Registration number and name of registry	Methods: Source of data and participants (P15-17)
	**29**	Where the full study protocol can be accessed	Methods: Source of data and participants (P15-17)<br>Data Availability (P20)<br>Code Availability (P20)
	**30**	Sources of funding and other support; role of funders	Acknowledgements (P20-21)
